# OliveCan: A Process-Based Model of Development, Growth and Yield of Olive Orchards

**DOI:** 10.3389/fpls.2018.00632

**Published:** 2018-05-09

**Authors:** Álvaro López-Bernal, Alejandro Morales, Omar García-Tejera, Luca Testi, Francisco Orgaz, J. P. De Melo-Abreu, Francisco J. Villalobos

**Affiliations:** ^1^Departamento de Agronomía, ETSIAM, Universidad de Córdoba, Córdoba, Spain; ^2^Centre for Crop Systems Analysis, Wageningen University & Research, Wageningen, Netherlands; ^3^Department of Agronomy, Institute for Sustainable Agriculture, Spanish National Research Council (CSIC), Córdoba, Spain; ^4^Departamento de Ciencias e Engenharia de Biossistemas, Instituto Superior de Agronomia, Universidade de Lisboa, Lisbon, Portugal

**Keywords:** carbon assimilation, crop model, *Olea europaea* L., SPAC model, water stress, water uptake

## Abstract

Several simulation models of the olive crop have been formulated so far, but none of them is capable of analyzing the impact of environmental conditions and management practices on water relations, growth and productivity under both well-irrigated and water-limiting irrigation strategies. This paper presents and tests OliveCan, a process-oriented model conceived for those purposes. In short, OliveCan is composed of three main model components simulating the principal elements of the water and carbon balances of olive orchards and the impacts of some management operations. To assess its predictive power, OliveCan was tested against independent data collected in two 3-year field experiments conducted in Córdoba, Spain, each of them applying different irrigation treatments. An acceptable level of agreement was found between measured and simulated values of seasonal evapotranspiration (*ET*, range 393 to 1016 mm year^-1^; RMSE of 89 mm year^-1^), daily transpiration (*E*_p_, range 0.14–3.63 mm d^-1^; RMSE of 0.32 mm d^-1^) and oil yield (*Y*_oil_, range 13–357 g m^-2^; RMSE of 63 g m^-2^). Finally, knowledge gaps identified during the formulation of the model and further testing needs are discussed, highlighting that there is additional room for improving its robustness. It is concluded that OliveCan has a strong potential as a simulation platform for a variety of research applications.

## Introduction

Olive orchards represent the main component of agricultural systems in many semiarid regions with Mediterranean climate, reaching 10.1 Mha worldwide in 2011 (FAOSTAT, 2014). In countries where the cultivation of this tree species is done in extensive areas, olive cropping systems have become of high relevance not only from an economic perspective, but also from an ecological one. Olive orchards have been traditionally cultivated at low planting densities under low-input rainfed conditions. However, the increase in the demand for oil of recognized and consistently high quality in recent years has triggered the development and adoption of farming techniques aimed to improve productivity, such as localized irrigation, fertigation and mechanical pruning and harvesting. As a result, traditional rainfed olive orchards (<200 trees ha^-1^) coexist nowadays with new intensive (250–850 trees ha^-1^) or super-intensive (1200–3000 trees ha^-1^) irrigated plantations. The rapid changes in olive farming have raised questions on the economic and environmental sustainability of the different olive cropping systems under present and future climate scenarios. Given that an olive orchard is a complex system, its quantitative study via modeling is a crucial step in understanding its behavior in response to climatic and management factors.

To our knowledge, Abdel-Razik (1989) was the first researcher to describe a model for estimating the productivity of olive orchards. The model describes the growth of different organs by simulating radiation interception, photosynthesis, respiration, and applying simple allocation rules, but it does not consider the effects of planting density, canopy structure or pruning, and many of its equations lack a consistent theoretical basis. Villalobos et al. (2006) proposed a simpler approach to estimate biomass production and yield in olive canopies, based on the concept of annual radiation use efficiency and partitioning coefficients, yet this approach does not give insight about the dynamics of the system, its response to climatic variables (besides solar radiation) or the effect of management. More recently, Morales et al. (2016) presented a mechanistic model of olive oil production in the absence of any biotic or abiotic stress, based on a three-dimensional model of canopy photosynthesis and respiration and dynamic distribution of assimilates among organs. However, water stress is the main limiting factor for biomass production in rainfed and deficit-irrigated olive orchards (Moriana et al., 2003; Iniesta et al., 2009).

Simulating the water balance of an irrigated olive orchard is a particularly challenging task as the trees are typically watered by point-source emitters that keep a small fraction of the surface frequently wet while the remaining area remains dry, unless it rains. This fact results in differences between these two soil areas in relation to soil water content, the water fluxes determining the water balance (i.e., runoff, drainage, redistribution along the soil profile, soil evaporation, and root water uptake) and root length density (Fernández et al., 1991). Therefore, traditional modeling approaches based on the use of the average soil water content can lead to large errors, besides giving a poor insight into the system. One alternative consists of using a two-compartment model that solves the water balance separately for each zone of the soil. In this regard, Testi et al. (2006) proposed a model capable of simulating potential transpiration, separately calculating runoff, drainage and soil evaporation from the wet and dry fractions of the soil surface under localized irrigation. The model was developed to determine the potential irrigation needs of olive orchards, so its use is unfortunately limited to unstressed conditions. Lately, García-Tejera et al. (2017a) have formulated a soil-plant-atmosphere-continuum (SPAC) model capable of calculating root water uptake from soils with spatially heterogeneous distributions of water content and root length densities. Such a model also discretizes the soil into different soil zones and layers and, for the canopy, it considers two leaf classes (i.e., sunlit and shaded). Furthermore, the model by García-Tejera et al. (2017a) provides estimates of gross assimilation (*A*), offering an opportunity to link the water and carbon balances of olive trees.

The goal of this study is to present and test a process-oriented model integrating existing knowledge on the growth and development processes of olive orchards and capable to account for the impacts of water stress, management and climate on their productivity, in the absence of nutrient deficiencies, diseases and pests. The model, hereafter named ‘OliveCan’ -which comes from ‘Olive Canopy-,’ was formulated using the models by Testi et al. (2006); Morales et al. (2016) and García-Tejera et al. (2017a) as starting point.

## Materials and Methods

### Model Description

This section provides an overview of the main features and processes within OliveCan. An in-depth description of the model, along with its equations and scientific rationale is given as Supplementary Material. The code of OliveCan was written in Visual Basic 6.0.

OliveCan is subdivided into three main components (Supplementary Figure S1) that are devoted to the computation of the water and carbon balances of the olive orchard and to simulate the impacts of some management operations. The water and carbon balance components are interdependent (i.e., each one needs data provided by the other) and both of them require information on soil traits and weather data.

Although most processes in the model run at daily time steps, others (i.e., root water uptake, photosynthesis, maintenance respiration and chilling accumulation) are computed over the diurnal course and integrated to yield daily values. The number of sub-day periods per day to be considered is customizable through a user-defined parameter (*N*). The meteorological input data required consist of daily values of the following variables: maximum and minimum air temperatures (*T*_max_ and *T*_min_, respectively), average vapor pressure (*e*_a_), solar radiation (*I*_G,D_) average wind speed (*U*) and precipitation (*P*). For those processes simulated at sub-day intervals, OliveCan incorporates routines that disaggregate the daily values of weather data into theoretical diurnal time series.

#### Water Balance Component

**FIGURE 1:**
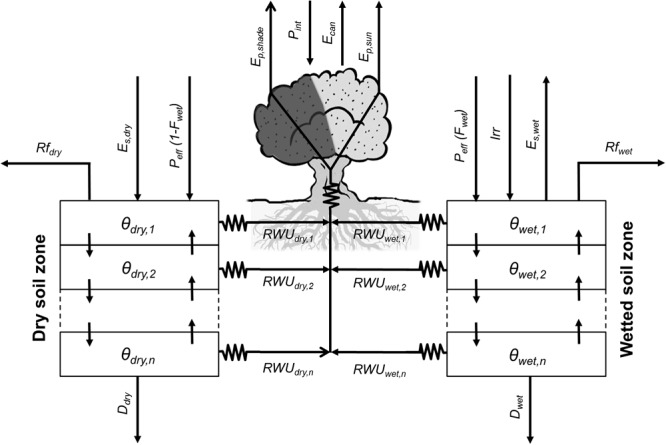
Relational diagram of the water balance component, highlighting the processes simulated. Solid arrows represent fluxes of water. In OliveCan, the water balance is solved separately for two soil compartments (i.e., dry and wetted soil zones), as irrigation (*Irr*) is applied to only one of them. For each soil zone, soil water content (i.e., 𝜃_dry_ and 𝜃_wetted_) are updated every day after calculating the fluxes of effective precipitation (*P*_eff,dry_ and *P*_eff,wetted_), runoff (*Rf*_dry_ and *Rf*_wetted_), drainage (*D*_dry_ and *D*_wetted_), water redistribution between the different soil layers, soil evaporation (*E*_s,dry_ and *E*_s,wetted_) and root water uptake (*RWU*_dry_ and *RWU*_wetted_). Direct evaporation of rain water intercepted by the canopy (*E*_can_) is also considered.

This modeling component simulates different physical and physiological processes relevant to olive water use (**Figure 1**). The model solves separately the water balance for two soil zones representing the dry and wetted (by localized irrigation) surface fractions. This approach enables the model to simulate the spatial heterogeneities in soil water content dynamics associated to the use of point-source emitters. Hence, the model considers that the water supplied by irrigation (*Irr*) is only applied to the wetted soil zone, whereas the dry soil zone is only watered by rainfall. On the other hand, runoff (*Rf*), soil evaporation (*E*_s_), root water uptake (*RWU*), and drainage (*D*) represent the water effluxes for both soil zones and are computed independently for each of those. Besides, each soil zone is subdivided into a user-defined number of soil layers (*n*) which are also customizable in thickness. Vertical soil water fluxes between adjacent soil layers are simulated, but no lateral flow between soil zones is considered.

Daily effective precipitation (*P*_eff_) is calculated by discounting rainfall interception by the canopy (*P*_int_) from total daily precipitation (*P*). *P*_int_ is calculated using a simplified version of the model of Gómez et al. (2001) and the resulting *P*_eff_ is distributed proportionally between the two soil zones as a function of the surface fractions that remain rainfed or are wetted by localized irrigation. With regard to *P*_int_, the canopy is treated as a capacitor capable of storing rain water up to a certain limit determined by canopy dimensions and leaf area index (*LAI*), according to Gómez et al. (2001). The stored water is subsequently lost by direct evaporation, which is simulated based on the Penman–Monteith equation assuming a null canopy resistance. As in Testi et al. (2006), the aerodynamic resistance is deduced from the model proposed by Raupach (1994), parametrized and validated specifically for olive orchards following Verhoef et al. (1997). The direct evaporation from wet foliage prevents tree transpiration (*E*_p_), until the intercepted water is totally lost.

Runoff and infiltration are calculated following a Soil Conservation Service curve number methodology that was specifically calibrated and validated for different typologies of olive orchards (Romero et al., 2007). The approach requires information on the canopy ground cover (*GC*) and the soil hydrological condition (*SHC*) -i.e., an indicative of the capacity of infiltration of the soil when it is wet. The water content at field capacity (𝜃_UL_), wilting point (𝜃_LL_) and saturation (𝜃_sat_) are also needed for the computation of infiltration and all the remaining simulated processes.

Drainage and soil water redistribution processes are simulated by CERES-type sub-models (Jones and Kiniry, 1986), while soil evaporation rates (*E*_s_) are estimated using the model of Bonachela et al. (2001). The latter, calculates *E*_s_ with a modified Penman-FAO equation for stage-one evaporation and uses the model of Ritchie (1972) for the soil-limited evaporation stage. For the wetted soil zone, microadvection effects are considered (Bonachela et al., 2001).

The model by García-Tejera et al. (2017a) is used to compute root water uptake (*RWU*) from each layer in the two soil zones, canopy transpiration (*E*_p_) and gross assimilation (*A*′). By analogy with the Ohm’s law for electric circuits, the model assumes that water transport through the SPAC is driven by differences in water potential and hydraulic resistances. In this regard, three hydraulic resistances are considered: from the soil to the root-soil-interface (*R*_s_), from the soil-root interface to the root xylem (*R*_r_) and from the root xylem to the canopy (*R*_x_). *R*_s_ depends on soil texture, root length density (*L*_v_), soil water content (𝜃) (Gardner, 1960). *R*_r_ is a function of *L*_v_ and root permeability, the latter being mediated by 𝜃 (Bristow et al., 1984) and temperature (García-Tejera et al., 2016). Finally, *R*_x_ is calculated from xylem anatomical traits and tree height. In the canopy, two leaf populations are considered (i.e., sunlit and shaded). For each one, gross assimilation (*A*′), stomatal conductance (*g*_s_), intercellular CO_2_ concentration (*C*_i_) and leaf water potential (Ψ_l_) are calculated iteratively, considering both the models by Farquhar et al. (1980) and Tuzet et al. (2003). In doing so, the environmental CO_2_ concentration (*C*_a_) is explicitly taken into account for calculating both *A*′ and *g*_s_ on the one hand. On the other, the model requires information on the intercepted photosynthetically active radiation (*IPAR*) as well as the sunlit and shaded fractions of the canopy. These inputs are provided by a simple geometric model of radiation interception which assumes a spheroidal shape for the crown and accounts for the shadowing from neighboring trees. Finally, *E*_p_ is estimated from the imposed evaporation equation assuming that the canopy is coupled to the atmosphere, whereas *RWU* is deduced in each layer of each soil zone from the corresponding water potential differences and hydraulic resistances.

#### Carbon Balance Component

**FIGURE 2:**
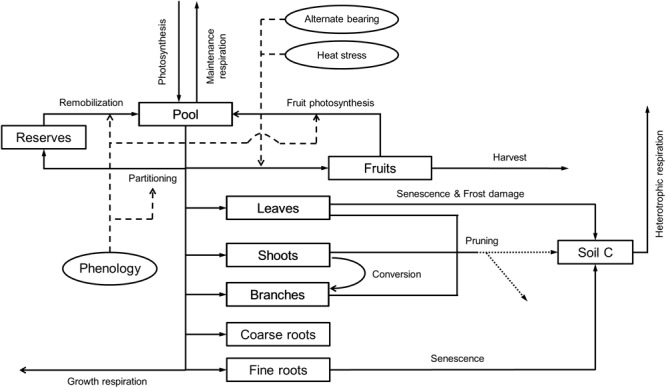
Relational diagram of the carbon balance component, highlighting the processes simulated. Squares represent carbon stocks and solid arrows the fluxes of carbon through the system. Circles and thin dashed arrows indicate key factors determining the calculation of some fluxes. Pruning residues can either be incorporated into the soil or exported, at user’s choice (dotted arrows).

This modeling component is aimed to simulate the growth and development of the trees and the carbon exchange of the orchard (**Figure 2**). First, the model calculates the daily pool of assimilates from the rate of *A*′ determined by the SPAC model. Based on experimental evidence, OliveCan also assumes that such pool can be increased to some extent by reserve remobilization (Bustan et al., 2011) or fruit photosynthesis (Proietti et al., 1999). The available assimilates are allocated to growth and respiration, the latter being segregated into maintenance and growth respiration (*RESP*_M_ and *RESP*_g_, respectively), of the different organs. In this regard, OliveCan considers six organ types: leaves, shoots (i.e., stems of up to 3 years-old), branches (including the trunk), coarse roots (i.e., roots with secondary growth), fine roots (i.e., absorbing roots) and fruits.

*RESP*_M_ is calculated as a function of temperature and biomass, and it is subtracted directly from the pool of assimilates. Whenever maintenance respiration exceeds the pool of assimilates, the deficit is discounted from the reserve pool. The remaining assimilates are distributed among the different organs with partitioning rules being mediated by phenology. The loss of carbon during the synthesis of new biomass was included by calculating a production value (*PV*) (Penning de Vries et al., 1974) for each type of organ according to its biochemical composition.

Two phenological stages are considered for the vegetative organs: (i) a dormant stage characterized by an absence of growth that is induced by chilling accumulation during autumn and (ii) a phase of active growth that starts in late winter, by the time average temperature is above a threshold. In relation to the reproductive growth, the date of flowering is determined with the two-phase model by De Melo-Abreu et al. (2004). Fruit growth is assumed to start after a given amount of thermal time is accumulated from the date of flowering and ceases when either maturity or the harvest date is reached.

During the vegetative rest period and provided that fruits are not present, all the available assimilates after discounting maintenance respiration are allocated to a virtual pool of reserves. Such reserve pool is subsequently used for the growth of vegetative organs and fruits during the growth season. Fruit growth can either be source-limited or sink-limited. In the former case, the associated partitioning coefficient is fixed whereas in the latter, it is calculated as a function of the number of fruits (*FN*), which in turn is modeled as a function of the number of fruits and nodes produced in the previous year. In doing so, the model may be prone to errors in the estimates of productivity and vegetative growth for a given year when performing long runs, but such errors are to be compensated if those model outputs are averaged over biennia. With regard to the vegetative organs, fixed partitioning coefficients are adopted. Whenever fruits are present, the model considers that they become the prioritary sink of assimilates, thus the vegetative partitioning coefficients are applied after discounting the fruit demand from the daily pool of assimilates. Therefore, partitioning coefficients to vegetative organs are assumed to be independent of tree size, management factors and environmental conditions, as in the model of Morales et al. (2016). As a final remark, inspired by the CERES-type models (Jones and Kiniry, 1986), the growth of fine roots is distributed among the different layers in the two soil zones as a function of the size and water content of each soil compartment.

Senescence of leaves and fine roots are simulated using a similar approach to that in the model by Morales et al. (2016). OliveCan takes also into account the conversion of shoots into branches when they exceed 3 years-old. Besides that, the model considers some of the effects of frost events and heat stress. Frost damage is simulated by assuming that a fraction of the standing leaves is defoliated when minimum air temperature falls below a certain temperature threshold. A similar approach is used for simulating the effect of extremely high temperatures during flowering on fruit set: when maximum air temperature exceeds a given threshold, a reduction in the final *FN* is triggered.

Variables related to canopy characteristics such as leaf area index (*LAI*) or *GC* are updated from the estimates of biomass of leaves assuming that the crowns present an spheroidal shape with constant leaf area density (*LAD*) and ratio of vertical to horizontal canopy radiuses (*R*_zx_). Similarly, the biomass of fine roots in each soil compartment is used to compute root length density (*L*_v_) by adopting a constant specific root length (*SRL*).

Finally, the soil carbon balance and heterotrophic respiration (*RESP*_H_) are computed with an adaptation of the model proposed by Huang et al. (2009) and modified to take into account the effect of soil moisture on the rate of decomposition according to Verstraeten et al. (2006). Then, by considering the different computed fluxes of assimilation and respiration within the orchard, OliveCan provides estimates of the ecosystem respiration (*RESP*_eco_) and net ecosystem exchange (*NEE*).

#### Management Component

Four management operations are considered in OliveCan: tillage, irrigation, harvest and pruning. In the model, tillage operations have an impact on *CN* whereas irrigation provides an additional water input for the wetted soil zone. Irrigation amounts and dates can either be defined explicitly by the users or implicitly calculated through a dedicated routine that, at customizable intervals, applies a fraction of the maximum *ET* lost since the last irrigation. Harvesting takes place on a user-defined day of the year and results in the removal of fruits. At harvest, the model provides an estimate of oil yield (*Y*_oil_) by multiplying the dry biomass of fruits and a fixed coefficient representing the ratio of oil content to dry matter. Finally, pruning is simulated by setting a customizable fraction of *LAI* to be removed (*F*_prune_) and an interval between pruning operations. The model also reduces the biomasses of shoots and branches by the same fraction *F*_prune_. The user should indicate whether pruning residues are incorporated into the soil or exported.

#### Initialization Requirements

Apart from the weather dataset and some orchard (e.g., planting density, age, and latitude) and soil (e.g., depth, 𝜃_UL_, 𝜃_LL_) basic traits, the user is required to enter the initial values of *GC* and *L*_v_ to deduce the biomasses of the different organs following simple criteria (see Supplementary Material). For the computation of *FN* in the first season, an estimate of dry yield for the year preceding the start of the simulation is also needed. To initialize the state variables related to phenology, simulations must start at the beginning of a year and the temperature records of the preceding 3 months must be provided. Some simulation settings such as the number of years to simulate and *N* must also be provided. Finally, the user is to indicate the management operations to be implemented and provide values to their parameters.

### Model Parameterization

When available, the values of the different parameters were taken from the literature. Supplementary Table S2 provides a complete list with the parameter values used for the simulations and the source from which they were taken. In short, the parameters of the SPAC model were taken from García-Tejera et al. (2017a,b), who, in turn, gathered most of the parameter values from different sources. Parameters related to phenology were obtained from reports by De Melo-Abreu et al. (2004) and López-Bernal et al. (2014, 2017). The studies by Mariscal et al. (2000) and Pérez-Priego et al. (2014) were used for setting the maintenance respiration and *PV* coefficients, respectively. Parameters related to the calculation of fruit number and yield were taken from several sources, including experimental data (see section “Number of Fruits and Alternate Bearing” in Supplementary Material). The coefficient of oil yield to dry fruit matter was taken from experimental data collected in a hedgerow cv. ‘Arbequina’ orchard (López-Bernal et al., 2015). Partitioning coefficients were based on findings by Mariscal et al. (2000); Villalobos et al. (2006) and Scariano et al. (2008). Reports from Barranco et al. (2005) and Koubouris et al. (2009) were used to parametrize the routines modeling the impacts of frost damage and heat stress, respectively. Coefficients modulating fine root growth distribution were directly taken from Jones and Kiniry (1986). Finally, parameters implied in the soil carbon balance were taken from Verstraeten et al. (2006); Huang et al. (2009) and, to a lesser extent, from other studies.

### Model Testing

Experimental measurements conducted in two mature olive orchards located in the Alameda del Obispo Research Station, Córdoba, Spain (37.8°N, 4.8°W, 110 m) were used for assessing the reliability of OliveCan. The climate in the area is typically Mediterranean, with around 600 mm of average annual rainfall and 1390 mm and average annual *ET_0_* of 1390 mm (Testi et al., 2004), respectively. The soil for both orchards is classified as a Typic Xerofluvent of sandy loam texture and exceeds 2 m in depth, with field capacity (𝜃_UL_) and permanent wilting point (𝜃_LL_) water contents of 0.23 m^3^ m^-3^ and 0.07 m^3^ m^-3^, respectively (Testi et al., 2004). Weather data were collected using a station placed 500 m away from the orchards. Within both orchards, irrigation experiments comprising several irrigation treatments were performed. Each irrigation treatment was simulated separately with OliveCan.

#### Experiment I

Extensive information on the orchard characteristics and dataset of Experiment I is provided by Iniesta et al. (2009). In short, the experiment was performed between 2004 and 2006 in a high density cv. ‘Arbequina’ olive orchard (tree spacing was 7 m × 3.5 m, i.e., 408 trees ha^-1^) planted in 1997. Irrigation was applied 5 days a week by drip, with seven emitters of 4 L h^-1^ per tree. A randomized complete-block design was used with three replications of 12 trees each, and the following irrigation treatments:

• Control irrigation (CON), which applied the required water to match the maximum *ET*, discounting rainfall. The maximum *ET* was estimated using the model of Orgaz et al. (2006).
• Continuous deficit irrigation (CDI), which applied 25% of the irrigation supplied to CON, distributed throughout the irrigation season.
• Regulated deficit irrigation (RDI), which applied the same seasonal water as CDI, with a midsummer (July 1st to September 10th–15th) deficit period without irrigation.

The measurements (only performed for the central trees of the replicates) used for testing the model were oil yield (*Y*_oil_, g m^-2^), seasonal *ET* and daily transpiration (*E*_p_, mm d^-1^). With regard to the former, each tree was manually harvested and the fresh yield weighed in the field. *Y*_oil_ was subsequently determined from sub-samples of 5 kg of fresh fruits. Cumulative *ET* was determined by water balance for the whole 2005 and 2006 seasons by measuring soil water content with a neutron probe (model 503, Campbell Pacific Nuclear Corp, Pacheco, CA, United States). Eight access tubes were installed between two trees per replicate, normal to tree rows. Measurements were taken at several depths (from 0.075 to 2.65 m deep). Finally, *E*_p_ was measured in 2006 with a sap-flow system device developed and assembled in the IAS-CSIC in Córdoba and described by Testi and Villalobos (2009). The system uses the Compensated Heat Pulse (CHP) method in combination with the Calibrated Average Gradient (CAG) procedure. The probes performed readings every 15 min at 4 depths in the xylem, spaced 10 mm. Six RDI, six CDI and four CON trees were instrumented with two probes per tree, at a height of 30 cm. The outputs of each probe were integrated first along the trunk radius and then around the azimuth angle. Average sap flow records for each treatment were calibrated against the estimates of *E*_p_ deduced from the difference between the measured *ET* and soil evaporation in a period of several weeks with no rainfall events during the summer. The model of Orgaz et al. (2006) was used to calculate soil evaporation. The calibrated sap flow data have not been published so far.

Values of *GC, LAD*, and *R*_zx_ required to initialize the model were taken from measurements of tree silhouettes. A record of *Y*_dry_ of the year preceding simulations was also considered. Initial *L*_v_ values were taken from records measured by Moriana (2001) for the trees of Experiment II.

#### Experiment II

Extensive information on the orchard characteristics and dataset of Experiment II is provided by Moriana et al. (2003). In short, the experiment was performed between 1997 and 1999 in a high density cv. ‘Picual’ olive orchard (tree spacing was 6 m × 6 m, i.e., 278 trees ha^-1^) of 18 years of age. Irrigation was applied 5 days a week by drip, with four emitters of 4 L h^-1^ per tree. A randomized complete-block design was used with three replications of 16 trees each, and the following irrigation treatments:

• Control irrigation (CON), which applied the required water to match the maximum *ET*, based on the fully replenishing soil water extraction from April to October.
• Regulated deficit irrigation (RDI), which applied 75% of the water received by CON (i.e., rainfall plus irrigation) with a midsummer deficit period (15 July to 15 September) without irrigation.
• Continuous deficit irrigation (CDI), which also applied 75% of the water received by CON (i.e., rainfall plus irrigation), but for the whole irrigation season.
• Alternate year irrigation (AYI), which was rainfed in the year of low crop load (1998) and fully irrigated, as CON, during the heavy crop years (1997 and 1999).
• Rainfed (DRY), which received no irrigation during the whole experiment.

The measurements (only performed for the central trees of the replicates) used for the model were *Y*_oil_ and seasonal *ET*. On the one hand, trees were harvested between December 15th and January 15th for the 3 years. Individual fruit weight of each tree was measured and a subsample of 150 fruits from each tree was used for determining oil content. On the other, cumulative *ET* was determined by water balance for each season by measuring soil water content with a neutron probe (model 503, Campbell Pacific Nuclear Corp, Pacheco, CA, United States). Eight access tubes were installed between two trees per replicate in the four irrigation treatments and six tubes were placed in the rainfed treatment. Measurements were taken were performed at several depths (from 0.075 to 2.4 m deep).

Values of *GC, LAD*, and *R*_zx_ required to initialize the model were taken from dedicated measurements. A record of *Y*_dry_ of the year preceding simulations was also considered. Initial *L*_v_ values were taken from records measured by Moriana (2001).

#### Statistical Analysis

Model performances in reproducing measured data were assessed using mean absolute error (MAE, from 0 to +∞, optimum 0), root mean square error (RMSE; from 0 to +∞, optimum 0), coefficient of residual mass (CRM, from -∞ to +∞, optimum 0) and modeling efficiency (EF, from -∞ to 1, optimum 1):

(1) $$\begin{array}{c} \mathrm{MAE}\;=\;\sum_{i}^{n}\left|S_{i}-M_{i}\right|/n \end{array}$$

(2) $$\begin{array}{c} \mathrm{RMSE}\;=\;\sqrt{\sum_{i}^{n}{\left(S_{i}-M_{i}\right)}^{2}/n} \end{array}$$

(3) $$\begin{array}{c} \mathrm{CRM}\;=\;1-\sum_{i}^{n}S_{i}/\sum_{i}^{n}M_{i} \end{array}$$

(4) $$\begin{array}{c} \mathrm{EF}\;=\;\frac{\sum_{i\;=\;1}^{n}{\left(M_{i}-\bar{M}\right)}^{2}-\sum_{i\;=\;1}^{n}{\left(S_{i}-M_{i}\right)}^{2}}{\sum_{i\;=\;1}^{n}{\left(M_{i}-\bar{M}\right)}^{2}} \end{array}$$

Where *M*_i_ is the *i*th measured variable, $\bar{M}$ is the average value of all measurements, *S*_i_ is the *i*th simulated variable and *n* is the number of measured values. In addition, the slope, intercept and coefficient of determination (*r*^2^) obtained by regressing the simulated and measured values were also used.

## Results

Measured and simulated values of *Y*_oil_ for the two experiments are also presented in **Table 1** in relation to the year and the irrigation treatment. Field measurements showed two common patterns, irrespective of the experiment. On the one hand, there were consistent differences in *Y*_oil_ between treatments, with the fully irrigated treatments (i.e., CON) exhibiting higher values than the deficit or rainfed ones. On the other hand, measurements revealed “high” *Y*_oil_ in the first and third experimental seasons and “low” *Y*_oil_ in the intermediate one. The only exception to this rule occurred in the second biennia of Experiment II for the DRY and RDI treatments, probably as a consequence of the high level of water stress reached in 1999 (Moriana et al., 2003). OliveCan reproduced well both the differences between treatments and the general alternating trend in *Y*_oil_ for both experiments. Pooling all the data together, MAE and CRM were close to zero, RMSE was 63.2 g m^-2^ and EF was 0.48. The regression analysis yielded an *r*^2^ of 0.51 and a slope of 0.65. Slightly better results in terms of regression parameters, RMSE and EF were found when analyzing the data grouped in biennia (**Table 2**).

**Table 1:** Observed (Obs.) and simulated (Sim.) values of seasonal evapotranspiration (*ET*) and oil yield (*Y*_oil_) in the model tests using the experimental data from Iniesta et al. (2009) (Experiment I) and Moriana et al. (2003) (Experiment II).

			*ET* (mm)		*Y*_oil_ (g m^-2^)	
Experiment	Treatment	Year	Observed	Simulated	Observed	Simulated
I	FI	2004	^∗^	798	319	255
		2005	958	862	151	119
		2006	1016	934	317	343
	CDI	2004	^∗^	697	247	241
		2005	621	612	131	115
		2006	709	672	267	243
	RDI	2004	^∗^	702	285	239
		2005	609	617	122	115
		2006	679	641	279	238
II	AYI	1997	822	642	357	254
		1998	576	509	21	91
		1999	839	643	244	191
	CON	1997	814	724	298	358
		1998	778	641	154	121
		1999	899	654	262	235
	DRY	1997	550	573	129	252
		1998	582	505	186	91
		1999	393	519	13	161
	RDI	1997	582	658	268	303
		1998	589	572	173	106
		1999	526	594	171	196
	SDI	1997	649	685	221	302
		1998	636	601	150	106
		1999	562	641	173	201

*^∗^Not measured.*

**Table 2:** Performances of OliveCan in reproducing the experimental data gathered for the model testing.

Process	Data	MAE	RMSE	CRM	EF	Slope	Intercept	*r*^2^
Seasonal *ET* (mm)	21	36.04	89.37	0.05	0.62	0.59	245.85	0.75
Daily *E*_p_ (mm)	1095	0.17	0.32	0.14	0.84	1.01	-0.19	0.90
Annual *Y*_oil_ (g m^-2^)	24	2.42	63.23	0.01	0.48	0.65	68.57	0.51
Biennial *Y*_oil_ (g m^-2^)	16	8.81	21.59	0.05	0.64	0.68	52.00	0.70

*MAE is mean absolute error, RMSE is root mean square error, CRM is coefficient of residual mass, EF is modeling efficiency and r^2^ is coefficient of determination.*

A good agreement was found between model estimates of seasonal *ET* and the measurements by Moriana et al. (2003) and Iniesta et al. (2009) (**Table 1**). Simulated values were close to experimental data and tracked reasonably well the differences between treatments. MAE and RMSE were 36.04 mm and 89.4 mm, respectively. The statistical analysis revealed no systematic bias, with CRM close to 0, and values of EF of 0.62 (**Table 2**). Nevertheless, the model was found to underestimate seasonal *ET* for the treatments receiving more irrigation, which led to a rather low slope for the linear regression fit (0.59) between simulated and observed values.

**FIGURE 3:**
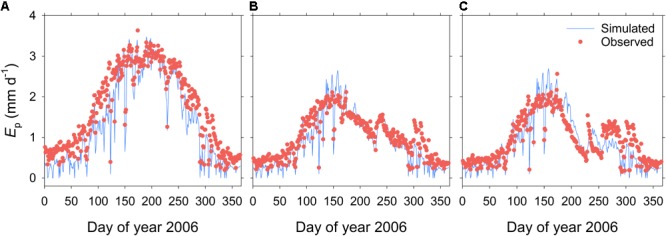
Time course of simulated and observed daily transpiration (*E*_p_) throughout the 2006 season for the three irrigation treatments of Experiment I: the fully irrigated control (CON) **(A)**, the continuous deficit irrigation (CDI) **(B)**, and the regulated deficit irrigation (RDI) **(C)** treatments.

Beyond the seasonal scale, OliveCan also provided good estimates of daily *E*_p_, as evidenced by comparing the model outputs with the calibrated sap flow measurements recorded in Experiment I (**Figure 3**). The model was found to slightly underestimate *E*_p_ in winter and late autumn, particularly for the CON treatment, which led to a CRM of 0.14. In addition, *E*_p_ was slightly overestimated during the RDI midsummer non-irrigated period. However, simulated and observed values were generally very close in the three treatments. Considering the three treatments together (*n* = 1095), the comparison between simulated and observed *E*_p_ values yielded a MAE of 0.17 mm d^-1^, a RMSE of 0.32 mm d^-1^, a EF of 0.84 and a satisfactory linear regression fit (**Table 2**).

**FIGURE 4:**
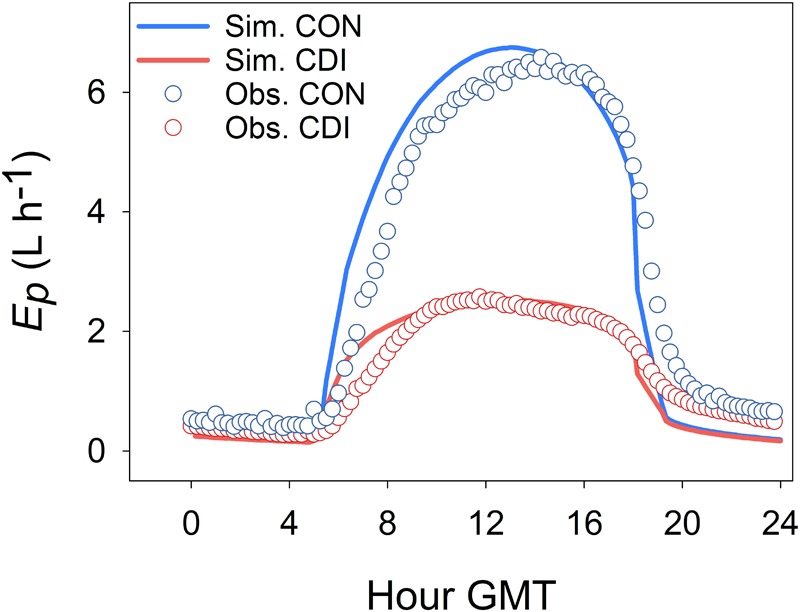
Daily course of simulated and observed transpiration (*E*_p_) in a typical sunny summer day (July 21st 2006, DOY 202) for the control (CON) and continuous deficit irrigation (CDI) treatments of Experiment I.

**Figure 4** illustrates the time course of measured and modeled *E*_p_ for the CON and CDI treatments over a typical sunny summer day of Experiment I (July 21st 2006, DOY 202). The four plotted *E*_p_ curves follow a bell shape, the CON ones exhibiting higher values than those of CDI. Apart from that, **Figure 4** shows a remarkable lag between the observed and the simulated daily course of *E*_p_, regardless of the treatment, with the diurnal trends of *E*_p_ being anticipated by the model. Hence, OliveCan tended to overestimate *E*_p_ in the hours following sunrise and to underestimate it around sunset.

## Discussion

### Model Performance

Model tests generally revealed a high level of agreement between simulations and experimental measurements. Given the variety of the simulated treatments and the many assumptions that a model like OliveCan must take, we found the results satisfactory. Notwithstanding that, there were situations in which model estimates departed from observations. For example, some discrepancies were found for some of the simulations of *Y*_oil_, *ET* (**Table 1**) and *E*_p_ (**Figure 3**), but, considering that the general trends and differences between treatments were captured by the model, we believe that the results are highly acceptable. Some of the divergences between measured and simulated *Y*_oil_ might be attributed to the fact that the approach followed by OliveCan to simulate alternate bearing is limited, as far as the physiological bases of alternate bearing are not completely understood yet (Connor, 2005; Dag et al., 2010). However, biennial comparisons (**Table 2**) only improved slightly the results. Apart from that, the remarkable lag between the simulated and measured diurnal courses of *E*_p_ (**Figure 4**) was to be expected: measurements were performed in the trunk with sap flow sensors and OliveCan does not simulate the buffering effect of the water stored in aboveground organs (Cermák et al., 2007). Also, the model assumes that stomatal conductance responds instantaneously to changes in environmental conditions, but the slow dynamics of stomatal opening and closing can cause lags in diurnal transpiration (Vialet-Chabrand et al., 2013).

Considering all the simulations together, the maximum simulated oil yield was 358 g m^-2^ (**Table 1**), which is comparable to the maximum values estimated by the model of Morales et al. (2016) and to available experimental data (Villalobos et al., 2006; Pastor et al., 2007). Simulated values of radiation use efficiency for oil production (i.e., the amount of oil produced per unit of intercepted PAR) averaged over biennia ranged between 0.17 and 0.10 g MJ^-1^. These estimates are within the range of variation found by Villalobos et al. (2006) across a wide range of commercial orchards in Southern Spain.

Overall, the results of all the aforementioned comparisons suggest that model performance is fairly satisfactory. However, further testing against experimental data taken from different environmental conditions and orchard characteristics seems highly desirable. This would help to provide additional evidence on the predictive power of OliveCan, or else to identify situations for which model accuracy could be improved through either better calibrations or reformulation of some routines. Apart from that, it should be noted that the reliability of OliveCan for estimating certain output parameters (e.g., *NEE, RESP_H_*) has not been tested specifically in the present study, which should also be the focus of future research efforts.

### Model Applicability

Considering its mechanistic approach, the vast quantity of simulated processes and its potential uses, OliveCan represents a momentous step forward in relation to previous olive growth simulation models. In this regard, OliveCan enables one to assess the combined effects of management operations and weather over crop performance for different olive orchard and soil typologies both under unstressed and water deficit conditions. Thus, the model shows potential for a broad range of research applications. For instance, OliveCan seems particularly suitable for assessing the performance of olive orchards under future climatic scenarios, as the model explicitly accounts for the multiple effects of reduced rainfall and increased environmental CO_2_ and temperature for the water and carbon balances of the orchard and the development of trees.

Obviously, the comprehensive nature and the wide range of simulated processes come at the expense of both model complexity and high input requirements. The latter is likely to be its main limitation, as far as some of the inputs (e.g., soil depth, *L*_v_ distribution) are not easy to measure in the field. In any case, it is noteworthy to emphasize that OliveCan has not been primarily conceived as a decision support system for farmers, but as a research tool.

### Further Research

During the development of the model, it became apparent that our current understanding of some of the physiological processes to be simulated was limited. For example, timing of vegetative bud break, dynamics of leaf senescence, fruit photosynthesis and the use of reserves are among the phenomena that have received less attention in the literature. Also, OliveCan is missing a sub-model aimed to properly simulate the dynamics of oil accumulation during the fruit growth period. Further research on these and other topics (e.g., alternate bearing) are clearly needed and might result in model improvements through either a more consistent parametrization or the formulation of better equations for simulating such processes.

Further research regarding genetic variability in model parameters is also desirable. With the exception of those related to the simulation of flowering date (De Melo-Abreu et al., 2004) and frost damage (Barranco et al., 2005), all parameters have been taken from past experiments carried out either with only one cultivar each (‘Arbequina’ being the most frequent) or averaging the results obtained for a few of them. Although the scarce literature does not allow us to disentangle how many of these crop parameters are cultivar-specific, it is clear that exploring their genetic variability might be important for enhancing model reliability. Moreover, the quantification of such cultivar variability may be used for evaluating its impact on tree physiology and productivity under different management, weather or orchard characteristics using OliveCan, which may be useful for breeding purposes.

Finally, future improvements of OliveCan might include additional sub-models for simulating nutrient uptake and the impact of pests and diseases. Apart from that, the model shows potential for being adapted to other tree species, so its interest may not be only restricted to olive researchers.

## Conclusion

The model presented here targets the simulation of the interactions between olive trees and their environment through a detailed characterization of the water and carbon balances of the orchard as affected by weather variables, soil attributes and management operations. The generally high level of agreement found between measured and simulated data evidence the suitability of OliveCan for estimating olive orchard dynamics. These results encourage the application of the model to simulate the growth, carbon exchange and water relations of olive orchards in a wide range of research contexts, including studies on the performance of olive trees under climate change scenarios. The development of OliveCan has also highlighted significant knowledge gaps in relation to some physiological processes and the cultivar specificity of some of the parameters. Further research on these aspects may contribute to improve the reliability of the model.

## Author Contributions

All authors played a significant role in the conception and development of the model. FV led out the coding, with contributions from ÁL-B, AM, OG-T, and LT. ÁL-B, LT, and FV gathered the datasets for testing the model. ÁL-B led out the writing with significant contributions from all co-authors.

## Conflict of Interest Statement

The authors declare that the research was conducted in the absence of any commercial or financial relationships that could be construed as a potential conflict of interest.
