# Supplementary material for: OliveCan: A Process-Based Model of Development, Growth and Yield of Olive Orchards
Source: Front Plant Sci. 2018 May 9;9:632. doi: 10.3389/fpls.2018.00632 (PMC5954587; doi:10.3389/fpls.2018.00632)
Supplement: Supplementary file 1 [file Data_Sheet_1.docx]

Supplementary Material

OliveCan: a process-based model of development, growth and yield of olive orchards

Álvaro López-Bernal*, Alejandro Morales, Omar García-Tejera, Luca Testi, Francisco Orgaz, Jose Paulo De Melo-Abreu, Francisco J. Villalobos

*** Correspondence:** Luca Testi: [lucatesti@ias.csi](mailto:lucatesti@ias.csi)c.es

# Supplementary Data

# Introduction

The aim of this supplementary material is to describe the model OliveCan. The model can be subdivided into three main components (Fig. S1) that are devoted to the computation of the water and carbon balances of the orchard and to simulate the impacts of management operations. This supplementary material provides details on the computation of such balances and the interactions between the components. Besides, this document provides the parameter values used in the simulations and the sources from which they were taken.

OliveCan is implemented as a discrete dynamical system (i.e. using difference equations instead of differential equations) with an intrinsic time-step of one day for most of its parts. However, some sections (i.e. the soil plant atmosphere continuum (SPAC) and radiation interception models, maintenance respiration and chilling accumulation) are computed over the diurnal course and integrated to yield daily values when required. The number of sub-day periods per day considered is a user-defined parameter (*N*). The input weather data required for sub-day calculations are obtained by disaggregation of daily values into theoretical diurnal time series.

# Water balance module

This is the first main component of OliveCan and it is devoted to simulate the water balance of both irrigated and non-irrigated olive orchards, computing the fluxes of effective precipitation, runoff, drainage, soil evaporation, root water uptake and direct evaporation of the rain water intercepted by the canopy. For irrigated orchards, the model computes the water balance separately for two soil zones: one watered by point-source emitters that keep a fraction of the surface (*F_wet_*) frequently wet and one representing the remaining fraction of the surface (1-*F_wet_*), which remains dry unless it rains.

## Precipitation interception

Precipitation may be intercepted by the canopy, reducing the effective amount of precipitation that reaches the soil surface. The model by Gomez et al. (2001) is used to calculate the fraction of incoming precipitation intercepted by the canopy. Accordingly, the maximum amount of precipitation that can be stored in a canopy (*Sto_max_*, mm) increases with the ratio between leaf area index (*LAI*, m^2^ leaf m^-2^ ground) and ground cover (*GC*, dimensionless):

${Sto}_{max}=0.49 \frac{LAI}{GC}+1.2$ (S1)

The amount of precipitation intercepted by the canopy increases exponentially with the daily amount of precipitation (*P*, mm d^-1^) up to the maximum storage:

$P_{int}={Sto}_{max}\left[ 1-\exp\left( -P/C_{fac} \right) \right]GC$ (S2)

where *C_fac_* (mm d^-1^) is an empirical coefficient which is also a function of *LAI* and *GC*:

$C_{fac}=2.214+0.55\frac{LAI}{GC}$ (S3)

Finally, the total amount of precipitation reaching the soil (*P_eff_*, mm d^-1^) is calculated as:

$P_{eff}=P-P_{int}$ (S4)

The effective precipitation reaching each soil zone is assumed proportional to the fraction of soil wetted by emitters (*F_wet_*):

$P_{eff,dry}=P_{eff}\left( 1-F_{wet} \right)$ (S5)

$P_{eff,wet}=P_{eff}F_{wet}$ (S6)

## Infiltration and runoff

Infiltration rates in each soil zone (*Inf_dry_* and *Inf_wet_*, both in mm d^-1^) are calculated as:

${Inf}_{dry}=P_{eff,dry}-{Rf}_{dry}$ (S7)

${Inf}_{wetted}=P_{eff,wet}+Irr-{Rf}_{wet}$ (S8)

Where *Rf_dry_* and *Rf_wet_* are runoff rates for each soil zone (mm d^-1^) and *Irr* is the amount of water applied by irrigation (mm d^-1^). In the following, the equations applied are exactly the same for both soil zones, so sub-indexes are avoided for the sake of simplicity. The underlying basis of such equations lies on the empirical curve number (*CN*) method (SCS-CN; Soil Conservation Service, 1985). For each zone, $Rf$ is calculated as:

$Rf=\left\{ \begin{matrix} 0 & P_{eff}\leq I_{a} \\ \frac{(P_{eff}-0.2 S_{max})^{2}}{P_{eff}+0.8 S_{max}} & P_{eff}>I_{a} \end{matrix} \right.$ (S9)

where *S_max_* (mm) is the maximum water depth that may be infiltrated or stored above the soil surface. The value of *S_max_* is calculated from the curve number (*CN*) as:

$S_{max}=254\left( \frac{100}{CN}-1 \right)$ (S10)

*CN* is a dimensionless empirical coefficient that varies between 0 and 100 and which depends on the properties of the soil surface. In OliveCan, *CN* is calculated from the model proposed by Romero et al. (2007) and validated for olive orchards. According to that model, *CN* is first determined assuming intermediate conditions of soil humidity (*CN2*, dimensionless).

Two procedures can be applied to obtain *CN2* depending on whether the soil is tilled or not. For bare soils under no tillage, the value of *CN2* is directly taken from tables reported in Romero et al. (2007) considering *GC* and the soil hydrological condition (*SHC*, dimensionless). S*HC* is an indicative of the infiltration rate of the soil when it is wet. In the model, *SHC* is treated as a parameter with integer values ranging between 1 (a category including clay and shallow soils, and soils with high phreatic level) to 4 (a category including sandy and depth soils with good drainage capacity).

For bare tilled soils, *CN2* is computed from three empirical coefficients *CN2F*, *CN2D* and *CPAR* and the accumulated rain kinetic energy (*SumE_rain_*):

$CN2=CN2D+\left( CN2F-CN2D \right)\exp\left[ -24.35 CPAR\cdot{SumE}_{rain} \right]$ (S11)

*CN2F*, *CN2S* and *CPAR* can be taken from tables as a function of *SHC* and *GC* (Romero et al., 2007). *SumE_rain_* increases with every precipitation. The kinetic energy (*E_rain_*) associated to a precipitation event is calculated as:

$E_{rain}=6.82P_{int}^{1.998}$ (S12)

Once *CN2* has been calculated, it is used to deduce the values of curve number for low (*CN1*) or high (*CN3*) soil humidity as:

$CN1=-4.7+0.89CN2$ (S13)

$CN3=3.3+1.05CN2$ (S14)

Then, *CN* is calculated as:

$CN=\left\{ \begin{aligned} CN2+\left( CN3-CN2 \right)CNPW \mathrm{if} CNPD>1 \\ CN2+\left( CN2-CN1 \right)CNPD \mathrm{if} CNPD <1 \end{aligned} \right.$ (S15)

$CNPW=\sum_{i=1}^{n} \frac{\theta_{i}-\theta_{UL,i}}{\theta_{sat,i}-\theta_{UL,i}}$ (S16)

$CNPD=\sum_{i=1}^{n} \frac{\theta_{i}-\theta_{LL,i}}{\theta_{UL,i}-\theta_{LL,i}}$ (S17)

where *θ_i_* is the water content of the layer “*i*” (m^3^ m^-3^), “*n*” is the number of soil layers and *θ_sat,i_*, *θ_UL,i_* and *θ_LL,i_* are, respectively, the relative soil water contents (m^3^ m^-3^) at saturation, field capacity and permanent wilting point.

## Drainage

The equations employed for the calculation of drainage rates (*D_dry_* and *D_wet_*, mm d^-1^) are the same for the two soil zones so, again, the corresponding sub-indexes are avoided for the sake of simplicity. The model computes *D* sequentially, starting with the upper layer that has received the precipitation/irrigation water as input and considering the values of *θ_sat,i_* and *θ_UL,i_*. These limits are compared to the actual water content in the soil layer (*θ_i_*):

$D_{i}=\left\{ \begin{matrix} 1000 \left( \theta_{i}-\theta_{sat,i} \right) {\Delta L}_{i} & \theta\geq\theta_{sat} \\ 1000 \left( \theta_{i}-\theta_{UL,i} \right) SWCON {\Delta L}_{i} & \theta_{sat}>\theta\geq\theta_{UL} \end{matrix} \right.$ (S18)

Where the factor 1000 accounts for the conversion of m to mm and *SWCON* is the fraction of soil water content above *θ_UL,i_* that drains on a period of one day and *ΔL_i_* is the width of each layer (m) in which the soil is divided. The values of *SWCON* depend on soil type (Villalobos et al., 2016a). The value of *D* for the last layer represents the actual drainage of the soil zone.

## Water redistribution

Water may also be redistributed in the soil following gradients of water potential. Water potentials are not explicitly calculated in the model at this stage but relative water contents (*RWC*, i.e. (*θ*-*θ_LL_*)/(*θ_UL_*-*θ_LL_*)) are used instead. Irrespective of the soil zone, the flow (*Flow*, mm) between two adjacent layers “*i”* and “*i+*1” is computed according to Ritchie (1998) as:

${Flow}_{i}=160\left[ \frac{{RWC}_{i}+{RWC}_{i+1}}{2}+0.5 \right]^{2}\frac{{RWC}_{i+1}-{RWC}_{i}}{0.5 \left( {\Delta L}_{i+1}+{\Delta L}_{i} \right)}$ (S19)

## Soil evaporation

The evaporation of water from the soil (*E_s_*, mm d^-1^) is calculated according to the model proposed by Bonachela et al. (2001). This is a modification of the frequently used two-stage soil evaporation model that takes into account that the wet fraction of the soil receives additional energy via convection from the air above the dry fraction of the soil (i.e. microadvective effect). The two stages considered in the model are:

1. When the soil surface is wet, soil evaporation is limited by the availability of energy and coincides with the potential evaporation as calculated by the Penman-Monteith equation. The stage does not finish until a given amount of water (*U_e_*, mm) has been evaporated.
2. When the upper layers of the soil dry, the diffusion of water vapour within the soil beings to limit the rate of soil evaporation, which becomes lower than (and decoupled from) the potential evaporation rate. The second stage becomes increasingly more influential with further drying of the soil.

Mathematically:

$E_{s}=\left\{ \begin{aligned} E_{s,pot} \mathrm{if} {CE}_{s}\leq U_{e} \\ c_{e}\left( t^{0.5}-\left( t-1 \right)^{0.5} \right) \mathrm{if} {CE}_{s}>U_{e} \end{aligned} \right.$ (S20)

Where *E_s,pot_* (mm d^-1^) is the potential soil evaporation (stage I), *CE_s_* is cumulative soil evaporation (mm), *c_e_* is a parameter that depends on soil type (mm d^-0.5^) and *t* is the time (d) since the start of stage II. The model takes into account that irrigation and precipitation will reduce the cumulative evaporation variable (${CE}_{s}$), potentially switching from stage II to stage I.

Although Eq. S20 remains the same for the two soil compartments, the model of Bonachela et al. (2001) calculates *E_s,pot_* differently to account for the microadvective effect. Hence, in the wetted soil zone, *E_s,pot_* is determined as:

$E_{s,pot,wet}=\left[ \frac{\Delta}{\Delta+\gamma}R_{n}\left( \frac{I_{s}}{I_{o}} \right)+\frac{\gamma}{\Delta+\gamma}VPD\cdot2.7\left( 1+\frac{U}{100} \right) \right]K_{sw}$ (S21)

Where *Δ* is the slope of the vapour pressure curve (kPa ºC^-1^); *γ*, the psychrometric constant (kPa ºC^-1^); *R_n_*, net radiation over grass (mm d^-1^); *I_s_/I_o_*, PAR transmissivity; *VPD*, vapor pressure deficit (kPa); *U*, wind speed at 2 m height (km h^-1^) and *K_sw_* is a microadvective coefficient, which can be estimated as:

$K_{sw}=1.1+0.14 \ln\left( 1/{F_{wet}} \right)$ (S22)

On the other hand, potential evaporation from the dry zone is:

$E_{s,pot,dry}={ET}_{0}\left( 1-Q_{d} \right)$ (S23)

where *ET_0_* represents the reference evapotranspiration (mm) and *Q_d_*, the fraction of intercepted photosynthetically active radiation (PAR, dimensionless). As indicated above, the former is calculated with the FAO Penman-Monteith method (Allen et al., 1998), whereas the latter is deduced from the radiation interception submodel described further on in this Supplementary Material.

## Canopy evaporation

The model solves a balance of the water stored in the canopy (*S_can_*, mm) every day. Such water appears as a consequence of the interception of rainfall (*P_int_*, mm) and is subsequently lost by direct evaporation (*E_can_*, mm):

$S_{can,t+1}=S_{can,t}+P_{int}- E_{can}$ (S24)

Where *S_can,t_* and *S_can,t_*_+1_ represent the water stored in the canopy in the days “*t*” and “*t*+1”. OliveCan calculates the evaporation of the water stored in the canopy based on the Penman-Monteith equation assuming a null canopy resistance. Such equation defines the potential direct evaporation from the wet foliage for a given day (*E_can,pot_*, mm d^-1^) as:

$E_{can,pot}=86400\frac{\Delta\left( R_{n}-G \right)+{\rho c}_{p} VPD G_{a}}{{2.45\cdot10}^{6} \left( \Delta+ \gamma\right)}$ (S25)

Where *G* is ground heat flux (J m^-2^ s^-1^), *ρc_p_* is the volumetric specific heat of air (1200 J kg^-1^ K^-1^)and *G_a_* is the aerodynamic conductance of the boundary layer of the canopy (mm s^-1^), which is deduced from the model proposed by Raupach (1994), calibrated and validated specifically for olive orchards according to Verhoef et al. (1997). Then, the actual direct evaporation from the wet foliage (i.e. *E_can_*) is assumed to be equal to:

$E_{can}=\left\{ \begin{aligned} E_{can,pot} \mathrm{if} E_{can,pot}\leq S_{can} \\ S_{can} \mathrm{if} E_{can,pot}>S_{can} \end{aligned} \right.$ (S26)

## Root water uptake and SPAC

OliveCan uses the multi-compartment SPAC model of García-Tejera et al. (2017a). Such model can be divided into three different parts: the ‘supply function’, the ‘demand function’ and a simple model of radiation interception. The first provides the basic equations for computing root water uptake (*RWU*, mm d^-1^) for every soil compartment whereas the second deals with the estimation of *A’* and the transport of water through trunk and branches for two leaf classes -shaded and sunlit- requiring data on radiation interception, which are supplied by the third. As a difference with respect to the rest of processes simulated in the water balance module, the SPAC model runs at sub-day intervals, providing estimates of both *RWU* for each soil compartment and *A’* and *E_p_* for each leaf class, and updating the soil water content of each soil compartment at the referred sub-day intervals.

### Supply function

**3.9.1.1 Water potential and unsaturated hydraulic conductivity of the soil**

In any soil compartment, soil water potential (*Ψ_s_*, kPa) and soil unsaturated conductivity (*k*, kg s^-1^ m^-1^ kPa^-1^) can be related to *θ* according to empirical relationships (Campbell, 1985):

$\Psi_{s}=\Psi_{e}\left( \frac{\theta_{sat}}{\theta} \right)^{b}$ (S27)

$k=k_{sat}\left( \frac{\theta}{\theta_{sat}} \right)^{2b+3}$ (S28)

Where *Ψ_e_* is the air entry water potential (kPa), *k_sat_* is the soil saturated conductivity (kg s^-1^ m^-1^ kPa^-1^) and *b* is a shape factor. These coefficients can be taken from the literature as a function of soil typology (e.g. Campbell and Norman, 1998).

**3.9.1.2 Soil hydraulic resistance to water movement**

The hydraulic resistance for the movement of water from the mid-distance between two adjacent roots towards the root surface (*R_s_*, kPa m^2^ s kg^-1^) is derived from the analytical solution proposed by Gardner (1960). The equation relates the difference in matric potential between the soil and the root surface to the flux of water that passes through the root under steady state conditions:


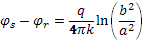
$\Psi_{s}-\Psi_{r}=\frac{q}{4\pi k}ln\left( \frac{{d_{root}}^{2}}{{r_{root}}^{2}} \right)$ (S29)

where *Ψ_r_* and *Ψ_s_* are the water potential at root surface and the soil water potential (kPa), *k* is the unsaturated soil water conductivity (kg s^-1^ m kPa^-1^), *q* is the water flux from the soil to a single root (kg m^-1^ s^-1^), *d_root_* is the half distance between roots (m) and *r_root_* is the root radius (m). *d_root_* is computed from root length density (*L_v_*, m root m^-3^ soil), assuming that roots are evenly distributed in the soil (Newman, 1969):


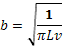
$d_{root}=\sqrt{\frac{1}{\pi L_{v}}}$ (S30)

Equation S29 can be rewritten to calculate *R_s_* (García-Tejera et al. 2017a):

$R_{s}={\ln\left( \frac{1}{\pi L_{v}{r_{root}}^{2}} \right)}/\left( 4 \pi k L_{v} \Delta L \right)$ (S31)

In the simulations, the value of *r_root_* (1.6 10^-4^ m) was taken from measurements performed by García-Tejera et al. (2017b) in an hedgerow olive orchard.

**3.9.1.3 Root radial resistance**

Total resistance in the radial direction from root surface to the root xylem (*R_r_*, kPa m^2^ s kg^-1^) is computed as:

$R_{r}={r_{r}}/\left( L_{v} \Delta L \right)$ (S32)

The value of *r_r_* (kPa m s kg^-1^) integrates, for a root section, the degree of permeability of the different tissues arranged in series that conform the root cylinder (Steudle and Peterson, 1998). In the present model, *r_r_* is obtained as a function of the temperature using the empirical relationship obtained by García-Tejera et al. (2016) and then modified according to *θ* using Bristow’s model (Bristow et al., 1984). The former relationship was estimated for 1-year old ‘Picual’ olive plants in the temperature range from 10 to 30 ºC:

$r_{rT}=64934.88+1.09\cdot{10}^{9} T^{-3.64}$ (S33)

where *T* is expressed in ºC. The model uses mean daily temperatures when applying the latter equation, as proposed by García-Tejera et al. (2016).

In order to account for the influence of water content, Bristow et al. (1984) proposed the following empirical correction:

$r_{r}=r_{rT}\left( 1+\alpha_{B}exp\left[ -\beta_{B}\left( \frac{\theta}{\theta_{sat}}-\delta_{B} \right) \right] \right)$ (S34)

Where *δ_B_, α_B_* and *β_B_* are empirical dimensionless parameters that are directly taken from Bristow et al. (1984).

**3.9.1.4 Tree collar water potential**

*E_p_* must be equal to the sum of *RWU* from all the layers in the two soil zones. Considering the soil compartment defined by layer “*i*” and zone “*j*”, *RWU_i,j_* might be deduced from the water potential difference between the soil (*Ψ_si,j_*) and the root xylem (*Ψ_rx,i,j_*) as well as the computed values of *R_s,i,j_* and *R_r,i,j_*. The integration of all those fluxes to obtain *E_p_* must be:

$E_{p}=\sum_{i=1}^{i=n} \sum_{j=dry zone}^{j=wetted zone} \frac{\Psi_{si,j}-\Psi_{rx,i,j}}{R_{si,j}+R_{ri,j}}$ (S35)

Eq. S35 can be further simplified. Unless cavitation is present, root xylem resistance is much smaller than *R_s_* and *R_r_* (Tyree and Zimmermann, 2002). Considering a negligible xylem resistance necessarily implies a common xylem water potential throughout the root xylem system. In other words, the water potential in the collar (*Ψ_c_*, kPa) is assumed to be the same in all the root vascular system. Under these circumstances, Eq. S35 can be rearranged as:

$\Psi_{c}=\frac{\sum_{i=1}^{n} \sum_{J=dry zone}^{j=wetted zone} \frac{\Psi_{s,i,j}}{R_{s,i,j}+R_{r,i,j}}-E_{p}}{\sum_{i=1}^{n} \sum_{J=dry zone}^{j=wetted zone} \frac{1}{R_{s,i,j}+R_{r,i,j}}}$ (S36)

### Demand function

**3.9.2.1 Transport of water through the xylem**

The theoretical specific conductivity (*k_te_*, kg m^-1^ kPa^-1^ s^-1^) of the xylem can be estimated by applying the equation of Hagen–Poiseuille:

$k_{te}=\frac{\pi\rho}{128 \eta A_{sap}}\sum_{i=1}^{n_{v}} \Phi_{v,i}^{4}$ (S37)

Where *ρ* and *η* are density and dynamic viscosity of sap, respectively (whose values can be set as those of the water: 1000 kg m^-3^ and 10^-6^ kPa s), *A_sap_* (m^2^) is the total sapwood cross-sectional area, *ϕ_v,i_* is the diameter of the *i*th xylem vessel (m) and *n_v_* the total number of vessels in the considered cross section (Tyree and Ewers, 1991). Given that olive wood presents a marked diffuse porosity, the equation can be simplified by considering the average vessel diameter (*ϕ_v_*, m) and vessel density (*VD*, vessels m^-2^ sapwood):

$k_{te}=\frac{\pi\rho}{128\eta}VD\phi_{v}^{4}$ (S38)

Inverting the latter equation, the model obtains the theoretical specific xylem resistivity (*r_te_*, m s kPa kg^-1^). Estimates of *r_te_* based on the equation of Hagen-Poiseuille are usually far below the values measured experimentally in wood segments because it does not account for the additional inter-conduit resistance arising from sap crossing a porous membrane to flow from one conduit to the next (Sperry et al. 2006). Hence, total hydraulic specific resistivity (*r_t_*, kg m^-1^ s^-1^ kPa^-1^) can be calculated as the sum of the lumen and inter-conduit resistances, as they are arranged in series:

$r_{t}=r_{te}+r_{pit}=\frac{1}{k_{te}}+r_{pit}$ (S39)

Some studies have estimated that, in the case of angiosperm species, *r_pit_* contributes 56 % to total xylem resistance regardless of wood porosity -ring or diffuse- (Wheeler et al., 2005; Hacke et al., 2006; Sperry et al., 2006). As a result, we may write:

$r_{t}=\frac{1}{K_{t}}=\frac{r_{te}}{1-0.56}=\frac{1}{0.44 K_{te}}$ (S40)

As a final step, the model computes the root-to-leaf hydraulic resistance (*R_x_*, kPa s kg^-1^) considering the pathway length between roots and shoots, which is assumed to be 0.75 times tree height (*H_tree_*, m), and the cross-sectional area of sapwood per m^2^ soil (*SWA*, m^2^ sapwood m^-2^ ground):

$R_{x}=\frac{0.75H_{tree}}{SWA}r_{t}$ (S41)

As an addition to the García-Tejera et al. (2017a) SPAC model, OliveCan estimates *SWA* as:

$SWA=HV LAI$ (S42)

Where *HV* is the Huber Value expressed in m^2^ sapwood m^-2^ leaf.

The values of the parameters in this section (*ϕ_v_*, *VD* and *HV*) were taken from measurements reported in the study of López-Bernal et al. (2010), who worked with mature ‘Arbequina’ olive trees. Such values were 3.6·10^-5^ m, 5·10^7^ vessels m^-2^ sapwood and 2.81 m^2^ sapwood m^-2^ leaf for *ϕ_v_*,*VD* and *HV*, respectively.

**3.9.2.2 Canopy transpiration and photosynthesis**

OliveCan considers that the canopy is discretized into two leaf classes (i.e. sunlit and shaded leaves) when upscaling gross assimilation (*A’)* and transpiration (*E_p_* )from the leaf to the canopy level. Hence, total *E_p_* (mm s^-1^) and *A’* (µmol CO_2_ m^-2^ leaf s^-1^) are calculated by adding the estimates of each leaf class:

$E_{p}=E_{p,sun}+E_{p,shade}$ (S43)

$A'={A'}_{sun}+{A'}_{shade}$ (S44)

The model relates *E_p_* to the stomatal conductance using a simplified version of the Penman-Monteith equation, assuming that the aerodynamic conductance is much higher than the stomatal conductance due to the “roughness” of the canopy surface (Jarvis and McNaughton, 1986; Villalobos et al., 2000; Orgaz et al., 2007):

${E_{p,sun}=g}_{co2,sun}1.6\frac{VPD}{P_{atm}}{LAI}_{sun}$ (S45)

${E_{p,shade}=g}_{co2,shade}1.6\frac{VPD}{P_{atm}}{LAI}_{shade}$ (S46)

Where *LAI_sun_* and *LAI_shade_* (m^2^ leaf m^-2^ ground) are the leaf area index of sunlit and shaded leaves, respectively, which are computed for every sub-day period as described below. *P_atm_* represents the atmospheric pressure (kPa) and it is calculated from the altitude of the orchard (*Alt*, m):

$P_{atm}=101.3-0.115 Alt$ (S47)

In the following, equations are analogous for the two leaf classes, so the use of the corresponding sub-indexes is avoided for the sake of simplicity.

Eqs. S45 and S46 can be further developed using the adaptation of Leuning’s model proposed by Tuzet et al. (2003), in which, *g_co2_* is related to leaf *A’*, the concentration of CO_2_ at the substomatal cavities (*C_i_*, µmol mol^-1^), the CO_2_ compensation point (*Γ*, µmol mol^-1^) and an empirical function (*f(ψ_l_)*) accounting for the effect of decreasing *ψ_l_* on stomatal closure:

$g_{co2}=g_{0}+\frac{m A'}{C_{i}-\Gamma}f\left( \Psi_{l} \right)$ (S48)

Where *g_0_* is the night-time conductance (i.e. for zero net CO_2_ assimilation) (µmol CO_2_ m^-2^ leaves s^-1^), *m* is a dimensionless proportionality factor between *A’* and stomatal conductance and *f*(*Ψ_l_*) is calculated as:

$f\left( \Psi_{l} \right)=\frac{1+exp\left( s_{f}\Psi_{f} \right)}{1+exp\left[ s_{f}(\Psi_{f}-\Psi_{l}) \right]}$ (S49)

Where *Ψ_f_* (kPa) is a reference leaf water potential which marks a *Ψ_l_* threshold value below which *g_co2_* is limited by leaf water status and *s_f_* is a factor modulating the rate of the reduction of *g_co2_* with decreasing *Ψ_l_*.

For each leaf class, *A’* is computed using the model of Farquhar et al. (1980), whose general equation can be written as:

$A^{'}=\frac{F_{1}\left( C_{i}-\Gamma\right)}{F_{2} C_{i}+F_{3}}$ (S50)

The coefficients *F_1_*, *F_2_* and *F_3_* vary depending on whether *A’* is limited by the kinetics of ribulose biphosphate (RuBP) carboxylase/oxigenase, Rubisco, (*A’_v_*, µmol CO_2_ m^-2^ leaves s^-1^) or by the potential rate of RuBP regeneration limited by the potential rate of NADPH production (*A’_q_*, µmol CO_2_ m^-2^ leaves s^-1^). In the former case, Eq. 50 turns to:

$A_{v}^{'}=\frac{V_{c,max}\left( C_{i}-\Gamma\right)}{C_{i}+k_{c}\left( 1+\frac{o_{i}}{k_{o}} \right)}$ (S51)

Where *V_c,max_* is the maximum activity of Rubisco (µmol m^-2^ leaf s^-1^), *o_i_* is the intercellular oxygen concentration, assumed constant and equal to 2.05x10^5^ µmol mol^-1^ and *k_c_* and *k_o_* are the Michaelis-Menten coefficients of Rubisco with respect to intercellular CO_2_ and O_2_ (mol mol^-1^). No mesophyll conductance has been taken into account, thus, the model assumes the same concentration for the intercellular spaces and the carboxylation sites of RuBP.

On the other hand, considering the limitation in RuBP regeneration by the potential rate of NADPH production, Eq. S50 results:

$A_{q}^{'}=\frac{J^{'}\left( C_{i}-\Gamma\right)}{{4C}_{i}+8\Gamma}$ (S52)

Where *J* is the potential rate of electron transport (µmol m^-2^ leaves s^-1^), which increases with the photosynthetic photon flux *Q* (µmol photon m^-2^ leaf s^-1^) according to the following expression:

$\theta_{F}J^{2}-\left( \alpha_{F} Q+J_{max} \right)J+Q \alpha_{F} J_{max}=0$ (S53)

Where *α_F_* (mol e^–^ (mol photon)^–1^) and *θ_F_* (dimensionless) are parameters describing the low-light quantum efficiency and the degree of curvature of the parabola, respectively. On the other hand, *J_max_* is defined as the maximum potential electron transport rate (µmol m^-2^ leaves s^-1^).

The parameters *V_c,max_, J_max_*, *k_c_* and *k_o_* are assumed to vary with temperature following the expression of Bernacchi et al. (2001):

$Parameter=exp\left( \frac{c-\Delta H_{a}}{R\left( T_{l}+273 \right)} \right)$ (S54)

Where *c* is a scaling constant, *ΔH_a_* is activation energy (J mol^-1^), *R* is the molar gas constant (8.31 J mol^–1^ K^–1^) and *T_l_* is leaf temperature (ºC). As the model consider the canopy fully coupled to the atmosphere air temperature is used as a surrogate of *T_l_*.

Farquhar et al. (1980) established that *A’* is to be the minimum value between those estimated by Eqs. S51 and S52:

$A^{'}=min\left\{ A_{v}^{'},A_{q}^{'} \right\}$ (S55)

Parameters in this section were taken from different sources. Those appearing in the modelling framework proposed by Farquhar et al. (1980) were taken directly from Díaz-Espejo et al. (2006) who worked with ‘Manzanilla’ olive trees. On the other hand, the value of *m* was defined from the relationships between assimilation and stomatal conductance reported by Moriana et al. (2002) for ‘Picual’ olive trees. The remaining parameters (*g_0_*, *Ψ_f_*, *S_f_*) were deduced from measurements in a cv. ‘Arbequina’ olive orchard performed by García-Tejera et al. (2017b).

**3.9.2.3 Coupling supply and demand**

OliveCan assumes that the role of tree capacitance is negligible, so *E_p_* can be directly computed as:

$E_{p}=\frac{\Psi_{c}-\Psi_{l}}{R_{x}}=\frac{\Psi_{s}-\Psi_{c}}{R_{s}+R_{r}}$ (S56)

If *Ψ_c_* from Eq. S36 is substituted in the above expressions and rearranged as a function of *Ψ_l_*, we can reach to:

$\Psi_{l}=\frac{\sum\sum\frac{\Psi_{s,i,j}}{R_{s,i,j}+R_{r,i,j}}}{\sum\sum\frac{1}{R_{s,i,j}+R_{r,i,j}}}-E_{p}\left( \frac{1}{\sum\sum\frac{\Psi_{s,i,j}}{R_{s,i,j}+R_{r,i,j}}}+R_{x} \right)$ (S57)

For a canopy discretized into sunlit and shade leaves:

$\Psi_{l,sun}=\frac{\sum\sum\frac{\Psi_{s,i,j}}{R_{si,j}+R_{ri,j}}}{\sum\sum\frac{1}{R_{si,j}+R_{ri,j}}}-g_{co2,sun}1.6\frac{VPD}{P_{atm}}0.018\left( \frac{\sum\sum\frac{1}{R_{s,i,j}+R_{r,i,j}}+R_{x}}{f_{sun}} \right){LAI}_{sun}$ (S58)

$\Psi_{l,shade}=\frac{\sum\sum\frac{\Psi_{s,i,j}}{R_{si,j}+R_{ri,j}}}{\sum\sum\frac{1}{R_{si,j}+R_{ri,j}}}-g_{co2,shade}1.6\frac{VPD}{P_{atm}}0.018\left( \frac{\sum\sum\frac{1}{R_{s,i,j}+R_{r,i,j}}+R_{x}}{f_{shade}} \right){LAI}_{shade}$ (S59)

Where the factor 0.018 converts moles of H_2_O into kg of H_2_O, the factor 1.6 converts conductance for CO_2_ to that of water vapour, *f_sun_* represents the ratio *LAI_sun_/LAI* and *f_shade_* is *1- f_sun_*. Both *f_sun_* and *f_shade_* are computed for every period of the daytime, as described in the radiation interception sub-model. Besides, the latter equations imply that each leaf class is sustained by a proportion of roots and trunk equal to *f_sun_* and *f_shade_*, respectively.

Values of *Ψ_l_* and *g_co2_* in each leaf class are computed in an iterative procedure using *C_i_* as a convergence criterion. Before describing the iterative procedure to obtain *g_co2_* and *Ψ_l_*, we need to derive the equation of maximum stomatal conductance for *CO_2_* for a non-limiting leaf water potential (*g_co2,max_*, µmol CO_2_ m^-2^ leaf s^-1^). To compute *A’*, the general diffusion function is used, which multiplies *g_co2_* by the CO_2_ mole fraction difference between the substomatal cavities (*C_i_*) and the air surrounding the leaf (*C_a_*, µmol mol*^-1^*) plus the leaf mitochondrial respiration (*R_d_*, µmol m^-2^ ground s^-1^):

$A^{'}=g_{co2}\left( C_{a}-C_{i} \right)+R_{d}$ (S60)

Combining the general expression to compute *A’* derived by Farquhar et al. (1980) and Eq. S68, the value of *g_co2max_* can be derived as:

$g_{co2max}=\frac{F_{1} \left( C_{i}-\Gamma\right)-R_{d}(F_{2} C_{i}+F_{3})}{(F_{2} C_{i}+F_{3})\left( C_{i}-C_{a} \right)}$ (S61)

The iteration starts with an initial value for *C_i_* set to 0.7*C_a_*. The initial value is introduced in Eq. S61 to compute *g_co2max_* and, then, *A’* is computed from Eq. S60 using *g_co2max_*. In the next step, *Ψ_l_* is calculated from Eqs. S58 and S59 and it is used to compute actual *g_co2_* from Eq. S48. Finally a new *C_i_* (*C_i,new_*, µmol mol^-1^) is calculated from:

$C_{i,new}=C_{a}-\frac{A^{'}-R_{d}}{g_{co2}}$ (S62)

If the convergence criterion is not satisfied, *C_i,new_* becomes *C_i_* and the loop starts again. The process is repeated until the difference between *Ci* and *C_i,new_* is less than 1 micromol mol^-1^. It is important to indicate that this iteration procedure is applied independently for each leaf class. Finally, once an equilibrium point for *C_i_* is attained, values for *E_p_* and *Ψ_c_* are then obtained from Eqs. S45, S46 and S36 and the rate of root water uptake for any soil compartment is deduced as:

${RWU}_{i,j}=\frac{\Psi_{s,i,j}-\Psi_{c}}{R_{s,i,j}+R_{r,i,j}}$ (S63)

As a final remark, OliveCan considers a null *E_p_* (and *RWU*) whenever there is direct evaporation in the canopy (as a result of precipitation interception). In other words:

${RWU}_{i,j}=\left\{ \begin{aligned} \frac{\Psi_{s,i,j}-\Psi_{c}}{R_{s,i,j}+R_{r,i,j}} if S_{can}=0 \\ 0 if S_{can}>0 \end{aligned} \right.$ (S64)

The model also considers that some *RWU* can still occur when the water stored in the canopy is depleted before the end of the day. To do so, the time for the stored water to be completely evaporated (*t_can_*, h) is calculated as:

$t_{can}={S_{can}}/\left( 3600 E_{can,pot} \right)$ (S65)

### Radiation interception

In this section, the amount of radiation intercepted by the canopy is calculated, as required by the model of photosynthesis and transpiration. The basic equations are described below, without going into the details of how these equations arise from the fundamental underlying geometric relationships.

All the tree crowns in the virtual orchard are assumed to have identical geometric features which can be approximated by a spheroid with horizontal radius *r_x_* (m) and vertical radius *r_z_* (m). The shadow of an spheroid projected on a horizontal plane is an ellipse of minor radius *r_x_* and major radius *r_z_ / cos*(*θ_z_*), where *θ_z_* is the zenith solar angle. Taking as reference the area delimited by the midpoints between consecutive trees, the area projected by each tree (*A_sh_*, m^2^) is:

$A_{sh}=F_{1}-F_{2}=\frac{\pi{r_{x}}^{2}U}{\cos\left( \theta_{z} \right)}-\frac{2{r_{x}}^{2}U}{\cos\left( \theta_{z} \right)}\left( \arccos\left( A \right)-A\sqrt{1-A^{2}} \right)$ (S66)

Where *F_1_* represents the area projected by the tree when it is isolated, *F_2_* is the area projected that is overlapped by first-order tree crown neighbours (within a row) and *U* and *A* are calculated as:

$U=cos\left( \sqrt{1+\left( \frac{r_{z}}{r_{x}} \right)^{2}\mathrm{ta}n^{2}(\theta_{z})} \right)$ (S67)

$A=min\left( \frac{D cos(\theta_{z})}{2r_{x}U},1 \right)$ (S68)

Where:

$D=\sqrt{D_{tree}D_{row}}$ (S69)

Where *D_row_* and *D_tree_* are the distances between the planting rows and between consecutive trees within each row, respectively. For *A*=1, no overlapping among the shadows of neighboring trees occurs and the projected shaded area coincides with *F_1_*. For values *A* > 1, the projected shadow within the reference surface decreases to the value *F_1_* − *F_2_* and overlapping occurs at the extremes of the shadows (Fig. S2). The overlapping area at each extreme is *F_2_*/2. Note that this calculation only takes into account first-order neighbors.

The average transmissivity of the canopy (*τ*, dimensionless) is calculated assuming exponential light extinction as described by Beer’s law. For an spheroid, this transmissivity becomes:

$\tau=2\frac{1-(1+A_{i})e^{-A_{i}}}{A_{i}^{2}}$ (S70)

Where:

$A_{i}=G(\theta_{z})LAD\frac{1.5V}{PEA(\theta_{z})}$ (S71)

Where *G(θ_z_)* is the so-called G-function that describes the ratio between the leaf surface projected on the plane perpendicular to the direction of incoming radiation and the actual leaf surface. *V* is the volume of the crown and *PEA*(*θ_z_*) is the projected envelope area of the crown on the plane perpendicular to the direction of incoming radiation. In the non-overlapping region of the shadow, total transmissivity is simply *τ*, whereas in overlapped regions, it becomes *τ^2^* as the beam of radiation crosses two canopies. Per unit of ground area, the transmissivity has to be weighted by the fraction of the reference area that is covered by the projected shadows:

$\tau_{t}=\frac{(F_{1}-2F_{2})\tau+F_{2}\tau^{2}}{A_{ref}}$ (S72)

The transmissivity per unit of ground area can be calculated for any solar zenith angle. Daily incoming radiation is disaggregated into a diurnal trend and separated into direct and diffuse radiation according to the algorithm proposed by Spitters et al. (1986) (Section 5.1). Within a tree crown, the different leaves are oriented in different directions and have different levels of exposure to the incoming radiation. In order to simplify the calculations, the canopy is stratified into two leaf populations:

- *LAI_sun_* = Leaf area index exposed directly to the sun.
- *LAI_shade_* = Leaf area index shaded from the sun.

The value of *LAI_sun_* is calculated according to the following expression:

$LAI_{sun}=\frac{Q_{dir}cos(\theta_{z})}{G(\theta_{z})}$ (S73)

Where *Q_dir_* (dimensionless) is the fraction of direct radiation intercepted by the canopy. *LAI_shade_* is deduced as:

$LAI_{shade}=LAI-LAI_{sun}$ (S74)

The values of intercepted radiation per unit of leaf area for each leaf population (*IPAR_sun_* and *IPAR_shade_*, W m^-2^) are calculated as:

${IPAR}_{sun}=\frac{IPAR_{dir}}{LAI_{sun}}+\frac{IPAR_{dif}}{LAI}$ (S75)

$IPAR_{shade}=\frac{IPAR_{dif}}{LAI}$ (S76)

# Carbon balance module

## Overview

The ‘carbon balance module’ of OliveCan simulates the carbon balance of the trees. The model considers that the tree contains six different types of organs: fruits, leaves, shoots, branches (including the trunk), coarse (structural) roots and fine (absorbing) roots. Besides, an additional virtual compartment is considered for the reserves. Prior to allocation to each organ, the model calculates the daily pool of available carbon for growth (*Pool*, g G m^-2^ ground d^-1^) from the balance of fluxes responsible for generation and maintenance respiration of the standing biomass (g G m^–2^ ground d^-1^):

$Pool=A^{'}-{RESP}_{M}+{REM}_{res}+A_{fruit}^{'}$ (S77)

Where *A*’ is the CO_2_ assimilation rate calculated by the ‘water balance module’ and integrated over the day, *RESP_M_* (g G m^–2^ ground d^-1^) is maintenance respiration, *REM_res_* (g G m^–2^ ground d^-1^) is the amount of reserves being re-allocated to growth and *A’_fruit_* (g G m^-2^ ground d^-1^) is the fruit CO_2_ assimilation.

Once *Pool* is computed, the available assimilates are allocated to the different organs, with the partitioning being based on the phenological phase. In the particular case of fruits, the partitioning can be constrained by the number of fruits (*FN*, fruits m^2^ ground), in whose calculation, the impacts of alternate bearing behaviour and heat stress are taken into account. Finally, the biomass of each organ is updated after computing the losses associated to senescence (affecting to leaves and fine roots), frost damage (on leaves) and the impacts of pruning and harvest (described in Section 4) as well as the conversion of shoots to branches.

## Phenology

### Introduction

Three phenological variables (*FlStage*, *FrStage* and *VStage*) are defined according to the type of organ (flowering buds, fruits and vegetative organs, respectively) which progress is being tracked. Each variable has multiple values representing the different phases of activity of each type of organ. The rules to calculate the duration of each phase are described below. The phases of each phenological variable are of relevance for the calculation of the partitioning coefficients of assimilates to each type of organ and the date of flowering.

### Flowering buds

The variable *FlStage* represents the different phenological phases that a flowering bud must undergo before flowering and its main purpose is to predict the date of flowering as a function of temperature. Following the model proposed by De Melo-Abreu et al. (2004), two phases must be considered for the correct prediction of flowering date of olive trees:

1. Vernalization (*FlStage* = 0): This phase begins at the beginning of either October (DOY = 274) or April (DOY = 91), depending on whether the latitude corresponds to the northern or southern hemisphere, respectively; and ends after a certain amount of low temperatures (*SumU*, h) has been accumulated.

2. Forcing phase (*FlStage* = 1): This phase starts after the end of the previous phase and ends once sufficient thermal time (*TT0*, ºC d) has been accumulated to induce flowering.

Accumulation of chilling hours (*U*) for a given hourly temperature is calculated by the following piece-wise linear function (De Melo-Abreu et al., 2004):

$U=\left\{ \begin{aligned} 0 T\leq0 \\ \frac{T}{T_{0}} 0<T\leq T_{0} \\ 1-\left( T-T_{0} \right)\frac{1-a}{T_{x}-T_{0}} T_{0}<T\leq T_{x} \\ a T>T_{x} \end{aligned} \right.$ (S78)

Where *T_0_* (°C) is the most effective temperature for accumulation of chilling hours, *T_x_* (°C) is the minimum temperature where the loss of chilling hours is maximum (i.e. any temperature higher than *T_x_* will result in the same loss of chilling hours) and *a* (h) is the maximum loss of chilling hours. In the second phase, the amount of thermal time accumulated in one day is assumed proportional to the average daily temperature minus the base temperature (*T_b_*, °C), assuming a null accumulation in case that average daily temperature is lower than *T_b_*. Flowering occurs when a given thermal time value (*TT0*, ºC d) has accumulated.

The use of a two-phase model implies that high temperatures during the vernalization period will compensate previous accumulation of chilling hours, postponing flowering. However, higher temperatures after the required amount of chilling hours have been accumulated will lead to earlier flowering dates.

The parameters necessary to calculate the phenology of flowering buds were taken directly from the values proposed by De Melo-Abreu et al. (2004).

### Fruits

Trentacoste et al. (2012) showed that fruit weight in olive trees increase in proportion to thermal time, so the phenology of fruits in OliveCan is simulated based on a thermal time approach. Two main phases for fruit development are considered:

1. No fruits are present or they are not a primary sink of assimilates (*FrStage* = 0). This period lasts from the date of maturity or harvest to fruit set. Fruit set occurs when sufficient thermal time has been accumulated from flowering. Therefore, two parameters are included: the total thermal time requirement from flowering to fruit set (*TT1*, ºC d) and the base temperature (*T_b_*, ºC).

2. Fruits are present and become the primary sink of assimilates (*FrStage* = 1). This period starts with fruit set and ends when either maturity or harvest date are reached. Maturity date occurs once sufficient thermal time (*TT2*, ºC day) has been accumulated since fruit set. Again, the amount of thermal time accumulated in one day is assumed proportional to the average daily temperature minus *T_b_*.

Lack of adequate data makes the parametrization of fruit phenology particularly challenging. For instance, thermal time requirements for reaching maturity appear to vary with fruit load and olive cultivar (Trentacoste et al., 2012). Therefore, further research aimed to improve the parametrization or the modelling of fruit maturity is clearly deserved. In our simulations, we set values of 300 and 2800 ºC d for *TT1* and *TT2*, respectively, adopting the same base temperature as for flowering buds (9.1 ºC, De Melo-Abreu et al., 2004). The value of *TT2* leads, in the conditions of Andalusia, to maturity occurring during the winter (which is also typical of such region). In any case, it should be noted that the *TT2* parameter is not expected to play a part in the simulations conducted for the present study, as harvest date was always set by autumn.

### Vegetative organs

The aboveground organs of olive trees do not appear to grow during the winter season in the Mediterranean region. Although root growth during this period has been reported (Palese et al., 2000; Scariano et al., 2008), quantitative data are lacking, so its magnitude cannot be calculated. As a result, OliveCan assumes that all vegetative organs stops growing during a certain period of the winter (*VStage* = 0) and are actively growing for the rest of the season (*VStage* = 1). Still, it is difficult to define an approach for simulating the transitions between both phenological phases due to the scarce literature on the topic. Hence, OliveCan uses empirical rules derived from experiments conducted with young cv. ‘Arbequina’ olive plants in different locations and seasons by López-Bernal et al. (2014; 2017).

In the model, the dormant period (*VStage* = 0) starts whenever sufficient chilling hours (*SumU_V_*, h) below a given threshold (*T_UV_*, ºC) have accumulated since the beginning of either October (DOY 274) or April (DOY 91), depending on whether the latitude corresponds to the northern or southern hemisphere, respectively. Growth resumption is computed by comparing the average daily temperature with a given threshold (*T_BB_*, ºC). Starting with the winter solstice, *VStage* switches to “1” whenever a number of days with average temperature above a *T_BB_* are accumulated (*SumDT_BB_*, d). Note that this approach implies both that warm winters may allow the trees to continue growing, as pointed out in the literature (e.g. Hartmann, 1953) and that photoperiod does not play a role for entering the winter rest state, as proved by López-Bernal et al. (2014). Parameter values are shown in Table S3.

## Maintenance respiration

Maintenance respiration is calculated at sub-day intervals, based on the study of Pérez-Priego et al. (2014), assuming a constant specific maintenance respiration for each type of organ and a temperature factor:

${RESP}_{M,i}=\sum_{j=1}^{N} \sum_{i} B_{i}{resp}_{M,i} F_{T}$ (S79)

Where *B_i_* (g DM m^–2^ ground) is the biomass associated to organ “*i*”, *resp_M,i_* (g C g^–1^ DM d^-1^) is the specific maintenance respiration coefficient of organ “*i*” at 25 ºC, *F_T_* is a temperature factor (dimensionless) which is calculated as:

$F_{T}=exp\left( \beta_{R,i}T \right)$ (S80)

Where *β_R,i_* is an organ-specific parameter and *T* denotes air temperature. In the case of fine and coarse roots, the mean daily air temperature is used irrespective of the sub-day period.

Pérez-Priego et al. (2014) measured maintenance respiration rates for fruits, leaves and woody stems of 3 year-old ‘Arbequina’ trees over a range of temperatures and fitted their observations to the previous sub-model, obtaining the values of *r_M,i_* and *β_R,i_* for those organs. We adopted directly such parameters values, assuming the same coefficients for shoots, branches and coarse roots. To our knowledge, no direct measurements of olive fine roots maintenance respiration rates have been reported so far. Abdel-Razik (1989) estimated maintenance respiration coefficients at 25 ºC for leaves, fruits and fine roots from nitrogen and mineral contents. Fine roots presented slightly lower but similar values of *r_M_* in relation to leaves, so we adopted the same parameter values (*r_M_* and *β_R_*) for these two organs.

In practice, applying Eq. S79 for woody organs (branches and coarse roots) with the aforementioned respiration coefficients measured by Pérez-Priego et al. (2014) usually leads to unreasonably high values of *RESP_M_* in mature trees. Measurements of *resp_M,branch_* by Pérez-Priego et al. (2014) were performed for young trees (3 year-old) but it is known that maintenance respiration of the inner layers of woody organs in mature trees is negligible (Ryan, 1990). In order to correct for this effect, the model considers a fraction of “active biomass” for respiration (*B_i,active_*, g DM m^-2^), which is computed as:

$B_{i,active}=\sum_{j=1}^{{j=Age}_{sapwood}} B_{i,j}$ (S81)

where *B_i,j_* is the biomass of the organ “*i*” (branches or coarse roots) that was generated “*j*” years before (*j*=1 would represent the biomass produced in the current year) while *Age_sapwood_* is the age at which wood becomes inactive. The value of the latter parameter has been inferred according to the radial profiles of sap velocity reported for olive trees in the literature (Fernández et al., 2001; Nadezhdina et al., 2007; López-Bernal et al., 2010). Such radial profiles are generally decreasing, with the highest velocities found in young wood, and zero or near-zero velocities for wood older than 5-6 years (López-Bernal et al., 2010). For the sake of simplicity Eq. S81 assumes that wood remains fully active, so we set *Age_sapwood_* to half the 6 years value reported in the aforementioned study.

Finally, it is worth noting that after integrating the maintenance respiration of the several organs, the resulting value might be higher than the amount of assimilates available from photosynthesis or reserve re-allocation. We assume that when that is the case, the excess of assimilates is exceptionally supplied from the reserve irrespective of the phenological stage.

## Reserve remobilization

There is evidence that olive trees accumulate reserves during winter and remobilize them for growth subsequently (Bustan et al., 2011). In OliveCan, two periods of reserve remobilization are considered. The first takes place in spring, when reserve reallocation is assumed to concentrate for a time window of duration defined by a thermal time limit (*TT_res_*, ºC d), starting with the onset of vegetative growth (i.e. when *VStage* switch to 1). During this period, the rate of reserve remobilization is calculated as:

${REM}_{res}={K1}_{res}\left( B_{G,res}-B_{G,res,crit} \right)$ (S82)

Where *K1_res_* (d^-1^) is a constant coefficient, *B_G,res_* is the biomass of reserves (g G m^-2^ ground) and *B_G,res,crit_* is the threshold value of reserve biomass below which there is no remobilization of reserves for growth. Such threshold is defined as a fixed fraction (10 %) of the active biomass in woody organs (i.e. branches and coarse roots):

$B_{G,res,crit}=0.1\left( B_{branch,active}-B_{croot,active} \right)$ (S83)

Similarly, reserve remobilization is also contemplated once fruits are present (i.e. when *FStage* switch to 1):

${REM}_{res}={K2}_{res}\left( B_{G,res}-B_{G,res,crit} \right)$ (S84)

Given the lack of information regarding reserve accumulation and remobilization, setting literature-based parameter values is impossible. We assumed a value of 0.0167 d^-1^ for both *K1_res_* and *K2_res_*, and a 100 ºC d time window for *TT_res_*.

## Fruit photosynthesis

For a large part of the period of fruit growth, Proietti et al. (1999) found that photosynthesis occurs in the fruits, resulting in a partial re-assimilation of the CO_2_ produced by respiratory processes in these organs (40−80 %). Unfortunately, to our knowledge, no other studies have paid attention to the photosynthetic capacity of olive fruits. In the presence of fruits (*FrStage* = 1), OliveCan computes the contribution of fruit CO_2_ gross assimilation (*A’_fruit_*, g G m^-2^ ground d^-1^) to the daily carbon budget as:

$A_{fruit}^{'}=P_{fruit}\cdot FA\cdot FN\cdot{DL}/{24}$ (S85)

Where *P_fruit_* represents the CO_2_ gross assimilation rate per unit of fruit surface during the daytime (g G m^-2^ fruit d^-1^), *FA* (m^2^ fruit^-1^) is the total surface area of fruits, *FN* (fruits m^-2^ ground) is the fruit number (Section 3.7) and *DL* (h) is the duration of the daytime period, which is calculated from the latitude of the orchard and the day of year.

Proietti et al. (1999) monitored the evolution of dry matter, fruit radius and photosynthesis -besides other traits- of olive (cv. ‘Leccino’) fruits along one season. According to their results, fruit photosynthetic capacity was high in the initial phase of fruit growth and decreased throughout the season, reaching negligible values at some time before the harvest date (which was apparently close to maturity, as revealed by the high oil content reported). Using weather data from the location of the experiment, we derived a polynomial relationship fitting *P_fruit_* to thermal time accumulation (using 9.1 ºC as the base temperature).

To deduce *FA*, OliveCan first calculates the average volume and radius of single fruits, which are treated as spheroids:

$V_{fruit}=\frac{B_{fruit}}{\rho_{fruit} FN}$ (S86)

$r_{fruit}=\sqrt[3]{\frac{3 V_{fruit}}{4 \pi c}}$ (S87)

$FA=4 \pi{r_{fruit}}^{2}\left( 0.35+0.65 c \right)$ (S88)

Where *B_fruit_* (g DM m^-2^ ground) is the total dry biomass of fruits, *ρ_fruit_* (g DM m^-3^) is the dry density of fruits and *c* is a parameter representing the ratio between the vertical and the horizontal fruit radiuses. In the model, *ρ_fruit_* is calculated with a piecewise linear relationship as a function of thermal time, which was derived again from the data reported by Proietti et al. (1999). *Ad hoc* measurements of the vertical and horizontal radiuses performed in 50 olive cv. ‘Arbequina’ fruits in mid-August 2016 were used to estimate the value of *c*.

## Partitioning of assimilates

The allocation of assimilates to each organ (*GG_i_*, g G m^-2^ ground d^-1^) is governed by different rules that depend on the phenological state of both fruits and vegetative organs:

- From harvest to the onset of vegetative growth (*VStage* = 0 & *FrStage* = 0): all assimilates available after covering maintenance respiration are allocated to reserves so that:

${GG}_{res}=Pool$ (S89)

- From the onset of vegetative growth to fruit set (*VStage* = 1 & *FrStage* = 0): as fruits are not present, all the assimilates are distributed between the vegetative organs through fixed organ-specific partitioning coefficients (*PC_i_*). In other words:

${GG}_{i}={PC}_{i} Pool$ (S90)

Where “*i*” can denote any vegetative organ (i.e. leaves, shoots, branches, coarse roots or fine roots).

- From fruit set to either harvest or the start of the dormant period (*VStage* = 1 & *FrStage* = 1): the available assimilates are first used to satisfy the fruit demand and then, the remaining carbon in the pool is distributed between the vegetative organs by applying the aforementioned fixed organ-specific partitioning coefficients. In mathematical terms:

${GG}_{fruit}={PC}_{fruit} Pool$ (S91)

${GG}_{i}=\left( 1-{PC}_{fruit} \right) {PC}_{i} Pool$ (S92)

Where the sub-index “*i*” denotes again any of the vegetative organs. Contrary to the partitioning coefficients to the vegetative organs, the one for fruits (*PC_fruit_*) is not always constant, but it is estimated as a function of assimilate availability (*Pool*) and fruit number (*FN*), as described in Section 3.7.

- From the start of the dormant period to harvest (*VStage* = 0 & *FrStage* = 1): the available assimilates are used to satisfy fruit demand. In case that carbon availability exceeds fruit demand, the remaining assimilates are allocated to reserves:

${GG}_{fruit}={PC}_{fruit} Pool$ (S93)

${GG}_{res}=\left( 1-{PC}_{fruit} \right) Pool$ (S94)

Information regarding the partitioning of assimilates may be obtained from different experiments with olive trees growing in pots or in the field. Villalobos et al. (2006) calculated the partitioning coefficients to the different aboveground organs in a high density orchard cv. ‘Arbequina’, by means of linear regressions between cumulative biomass of the different organs between successive destructive harvests. The fractions allocated to leaves, structural branches and fruits over an entire year were 0.16, 0.34 and 0.50, respectively. Mariscal et al. (2000) reported in 1-year old olive trees partitioning coefficients of 0.24, 0.5 and 0.26 for leaves, structural branches and roots, respectively. Scariano et al. (2008) reported for 3-year old potted olive trees a partitioning of 0.31 to roots (0.20 to structural roots, 0.11 to fine roots), 0.28 to leaves and 0.41 to branches (no allocation to fruits was reported, which explains the higher values compared to the results from Villalobos et al., 2006). In relation to the partitioning coefficients reported by Scariano et al. (2008), it is important to realize that the ratio of biomass between fine and coarse roots measured in an experiment does not represent the relative allocation to these different organs, due to senescence of fine roots. Thus, the allocation to fine roots should be increased to compensate this loss. The model is expected to accumulate increasingly more biomass in the coarse roots whereas the fine root biomass reaches a maximum, determined by the balance of root production and senescence. On the other hand, the partitioning coefficients obtained by Villalobos et al. (2006) were defined at the annual level. The final choice of parameter values was made after considering all the above and it is shown in Table S2.

## Number of fruits and alternate bearing

Whenever present, fruits become the main sink of assimilates in the carbon balance. In OliveCan, the partitioning coefficient to fruits (*PC_fruit_*) is constrained by either the availability of assimilates or by the number of fruits (*FN*, fruits m^-2^ ground):

${PC}_{fruit}=\min\left( {PC}_{fruit,max}, \frac{{FGR}_{max} FN}{{PV}_{fruit} Pool} \right)$ (S95)

Where *PC_fruit,max_* is the maximum partitioning coefficient to fruits; *FGR_max_* (g DM d^-1^), the maximum fruit growth rate and *PV_fruit_* (g DM g^-1^ G), the ratio of dry matter to glucose equivalents for fruits; the three being parameters in the model.

The model computes *FN* as the product of the potential number of fruits (*FN_max_*, fruits m^-2^ ground) by a factor that depends on both the actual and maximum fruit number of the previous year (*FN_1_* and *FN_max,1_*, respectively):

$FN={FN}_{max}\left( 1-C_{alt} \frac{{FN}_{1}}{{FN}_{max,1}} \right)$ (S96)

Where *C_alt_* is a parameter accounting for the alternate bearing behavior that may be defined as the fraction of fruit positions that are lost following a season for which fruit load was maximum. *FN_max_* is calculated as a function of the leaf biomass generated in the precedent year (*W_leaf,1_*, g DM m^-2^ ground):

${FN}_{max}=\frac{W_{leaf,1}SLA}{2A_{leaf}}F_{FP}$ (S97)

Where *SLA* (specific leaf area, m^2^ leaf g^-1^ DM), *A_leaf_* (mean area of the leaves, m^2^ leaf) and *F_FP_* (ratio of fruits per leaf pair, dimensionless) are parameters.

Besides the aforementioned equations, *FN* can be further reduced by the occurrence of high temperatures around flowering as described in Section 3.12.

With regard to the parameters in this section, *PC_fruit,max_* was set to the maximum value (1) after considering together the studies of Villalobos et al. (2006) and Morales et al. (2016). The values of *A_leaf_* and *SLA* were taken from data by Villalobos et al. (2006).

The remaining parameters were determined from an unpublished two-year experiment in a four-year-old olive cv. ‘Arbequina’ orchard located in the Alameda del Obispo Research Station, Córdoba, Spain (37.8ºN, 4.8ºW, 110 m). Two treatments were considered, consisting of either removing or not 50 % of the inflorecences by June 1^st^ on the first experimental season. There were six replicates per treatment, with all the trees being fully-irrigated. *F_FP_* was determined during the thinning process and *FGR_max_* was specifically estimated from subsequent samplings in the fruit-thinned treatment. *C_alt_* was deduced from dedicated measurements in the two seasons for marked shoots in the non-thinned control.

## Growth respiration

Growth respiration (*RESP_g_*, g DM m^-2^ ground d^-1^) is calculated for each organ as:

${RESP}_{g,i}={GG}_{i} \left( 1-{PV}_{i} \right)$ (S98)

Where the sub-index “*i*” can denote any organ (i.e. fruits, leaves, shoots, branches, coarse roots or fine roots) and *PV_i_* (g DM g^-1^ G) is the efficiency by which assimilates are converted into dry matter of organ “*i*”. Hence, the daily growth of the organ “*i*” (*GB_i_*, g DM m^-2^ ground d^-1^) can be expressed as:

${GB}_{i}={GG}_{i}-R_{g,i}={GG}_{i} {PV}_{i}$ (S99)

Production value coefficients for leaves, shoots and branches were taken from reports in Mariscal et al. (2000), who worked with ‘Picual’ plants. *PV_croot_* was assumed equal to *PV_branch_* and *PV_froot_*, the same as *PV_shoot_*.

## Senescence

Reports in the literature indicate that the seasonal trends of leaf shedding in olive trees might peak at some specific periods of the year (Priestley, 1977; Abdel-Razik, 1989; Proietti, 1998) but the available data does not allow to define a clear annual distribution of leaf senescence. In the absence of adequate quantitative data, we assume in the model that leaf senescence is spread uniformly throughout the year and that the daily rate of leaf loss is constant (i.e. every day of the season the same amount of leaf biomass is lost due to senescence) and equal to the corresponding fraction (i.e. 1/365) of half the leaf biomass accumulated between the second and third years before, assuming an average leaf lifespan of 2.5 years for leaves. In mathematical terms, the daily rate of leaf senescence (*S_leaf_*, g DM m^-2^ ground) can be expressed as:

$S_{leaf}=\frac{1}{365}\left( 0.5B_{leaf,2}+0.5B_{leaf,3} \right)$ (S100)

Where *B_leaf,2_* and *B_leaf,3_* represent the total leaf biomass generated two and three years before, respectively.

No data was found regarding the senescence of fine roots in olive trees. We adopted a life span of one year and assumed again a constant daily senescence rate (*S_froot_*, g DM m^-2^ ground) along the year:

$S_{froot}=\frac{1}{365}B_{froot,1}$ (S101)

With *B_froot,1_* being the total fine root growth accumulated in the previous year. In the model *S_froot_* is both computed independently for each single soil compartment and then integrated over the different layers and soil zones.

## Conversion of shoots into branches

In OliveCan, a “shoot” is defined as any stem below three years of age. Therefore, the daily rate of conversion of shoots into branches (*BC*, g DM m^-2^ ground d^-1^) is calculated as:

$BC=\frac{1}{365}B_{shoot,3}$ (S102)

Where *B_shoot,3_* is the total shoot biomass produced three years before. The 1/365 factor implies that the model assumes a steady rate of conversion along the whole year.

## Frost damage

In the case of olive trees, usual frost damage symptoms include leaf chlorosis and defoliation (Barranco et al., 2005). OliveCan simulates the fraction of leaf area which is shed by frost damage (*F_leaf_*, dimensionless) following a piece-wise linear function:

$F_{leaf}=\left\{ \begin{aligned} 0 if T_{min}>T_{NF} \\ \frac{T_{min}-T_{CD}}{T_{ND}-T_{CD}} if T_{ND}\geq T_{min}\geq T_{CD} \\ 1 if T_{min}<T_{CD} \end{aligned} \right.$ (S103)

Where *T_min_* (ºC) is the minimum daily temperature, *T_ND_* (ºC) is the temperature threshold below which low temperatures result in defoliation and *T_CD_* (ºC) is the temperature below which the tree undergoes complete defoliation.

The values of the two temperature thresholds *T_ND_* and *T_CD_* were deduced from Barranco et al. (2005). These authors estimated *T_CD_* for several olive cultivars (for the cv. ‘Arbequina’ a value of -11.8 ºC was reported) from measurements of electrical conductivity in leaf samples after exposure to different sub-zero temperatures. In addition, in the same study it was measured the fraction of dry biomass of leafed shoots that were frozen in field trees of several cultivars after an event of -10.5 ºC. Using such fraction (0.626 for cv. ‘Arbequina’ trees) as a surrogate of *F_leaf_*, Eq. S103 was inverted to deduce the value of *T_ND_*, yielding -9.7 ºC.

## Heat stress

In the olive tree, some reproductive processes such as pollen dispersion, pollen germination, pollen tube growth and fruit set are known to be affected by high temperatures (Cuevas et al., 1994; Koubouris et al., 2009). Unfortunately, interactions with other climatic factors (e.g. relative humidity), differences between olive cultivars and scarcity of published reports on this topic prevents the establishment of accurate temperature thresholds for assessing the impacts of high temperatures on such processes.

In the model, a “heat-sensitive time window” around the date of full flowering is defined based on thermal time accumulation since the beginning of the forcing phase (*FlStage* = 1). Two parameters, *TT_HS1_* and *TT_HS2_* (ºC d^-1^), identify the beginning and end of the sensitive time window, which cover the whole flowering period plus some days before and after it. Values for *TT_HS1_* and *TT_HS2_* were fitted to ensure that this condition is met based on usual observations on the duration of flowering (values reported in Table S3).

During the sensitive time window, the model considers that heat stress events reduce the number of fruits produced (*NF*) by multiplying it by a factor (*F_fruit_*) which is calculated as:

$F_{fruit}=\prod_{Day when TT={TT}_{HS1}}^{Day when TT={TT}_{HS2}} F_{heat}$ (S104)

Where *F_heat_* is calculated daily as:

$F_{heat}=\left\{ \begin{aligned} 1 if T_{max}<T_{NH} \\ \frac{T_{CH}-T_{max}}{T_{CH}-T_{NH}} if T_{NH}\leq T_{max}\leq T_{CH} \\ 0 if T_{max}>T_{CH} \end{aligned} \right.$ (S105)

Where *T_NH_* represents the maximum daily temperature below which fruit number is not affected and *T_CD_* the maximum daily temperature threshold above which fruit production is completely inhibited. Koubouris et al. (2009) observed that pollen germination and tube growth was optimal between 20 ºC and 30 ºC and almost completely inhibited after 24 h exposure to 40 ºC. Although the results of such laboratory experiments are difficult to translate into the field, they at least suggest that *T_NH_* might lay somewhere between 30 and 40 ºC and *T_CH_* might be higher than 40 ºC. Thus, we adopted values of 35 ºC and 45 ºC for *T_NH_* and *T_CH_*, respectively.

## Updating state variables

The state variables of model are updated each simulated day by following difference equations:

$B_{fruit,t+1}=B_{fruit,t}+{GB}_{fruit}$ (S106)

$B_{leaf,t+1}=B_{leaf,t}+{GB}_{leaf}-S_{leaf}-F_{leaf,t} B_{leaf,t}$ (S107)

$B_{shoot,t+1}=B_{shoot,t}+{GB}_{shoot}-{CB}_{shoot-branch}$ (S108)

$B_{branch,t+1}=B_{branch,t}+{GB}_{branch}+{CB}_{shoot-branch}$ (S109)

$B_{croot,t+1}=B_{croot,t}+{GB}_{croot}$ (S110)

In the case of fine roots, the daily increase in fine root biomass (i.e. *GB_froot_*) is distributed among the different soil layers and zones, using an approach inspired by the CERES-type models (Jones and Kiniry, 1986). To do so, the model first checks if the whole soil profile has been explored by roots. If that is not the case, root depth (*Z_root_*, m) increases as a function of thermal time (*TT*, ºC d):

$Z_{root,t+1}=Z_{root,t}+\varphi{TT}_{t}$ (S111)

Where *φ* is a coefficient representing the daily increase in root depth per accumulated degree day (m ºC^-1^ d^-1^) and the sub-indexes “*t*” and “*t+1*” refer to two consecutive days. On the other hand, *TT* is computed as the average daily temperature minus the base temperature, *T_b._*

In a second step, the model calculates weigh factors for each soil layer explored by roots in each soil zone by considering its thickness and relative water content (*θ_rel_*, dimensionless). For a layer “*i*” and the soil zone “*j*”, the latter is defined as:

$\theta_{rel,i,j}=\left( \theta_{i,j}-\theta_{LL,i,j} \right)/\left( \theta_{UL,i,j}-\theta_{LL,i,j} \right)$ (S112)

The model considers that root growth is limited below a given value of *θ_rel_* (*θ_crit_*, dimensionless) and completely hindered at the permanent wilting point. Therefore, an initial weigh factor for the layer “*i*” in the zone “*j*” (*WF_i,j_*, dimensionless) is calculated as:

${WF}_{i,j}=\left\{ \begin{aligned} {\Delta L}_{i,j} \mathrm{if} \theta_{rel,i,j}\geq\theta_{crit} \\ 4\theta_{rel,i,j}{\Delta L}_{i,j} \mathrm{if} \theta_{crit}>\theta_{rel,i,j}>0 \\ 0 \mathrm{if} \theta_{rel,i,j}=0 \end{aligned} \right.$ (S113)

Such weigh factor must be subsequently corrected by taking into account the values in every soil compartment:

${WF}_{i,j}={{WF}_{i,j}}/{\sum_{i=1}^{i=nx} \sum_{j=dry}^{j=wetted} {WF}_{i,j}}$ (S114)

Where *nx* denotes the number of layers explored by roots.

Finally, the biomass of fine roots in any soil compartment is updated as:

$B_{froot,i,j,t+1}=B_{froot,i,j,t}+{GB}_{froot,t}{WF}_{i,j,t}-S_{froot,i,j,t}$ (S115)

With regard to reserves, their biomass (in glucose equivalents terms) is daily updated as:

$B_{G,res,t+1}=B_{G,res,t}+Pool\left( 1-{PC}_{fruit}-{PC}_{leaf}-{PC}_{shoot}-{PC}_{branch}-{PC}_{croot}-{PC}_{froot} \right)-R_{res}$ (S116)

As a final remark, harvest and pruning lead to losses in the biomass of fruits, leaves, shoots and branches that are not considered in these equations. The impacts of such operations into the carbon balance are described in Sections 4.3 and 4.4. The parameters *θ_crit_* and *φ* were directly taken from Jones and Kiniry (1986).

## Updating derived state variables

*LAI*, *GC*, canopy volume (*V*, m^3^), the horizontal canopy radius (*r_x_*) and *L_v_* are calculated from the biomass in leaves and fine roots. This section describes the basic equations used to update these variables from *B_leaf_* and *B_root_*.

Starting with the canopy-related state variables, *LAI* is calculated from *B_leaf_* as:

$LAI=B_{leaf} SLA$ (S117)

Then, the volume of the canopy (*V*, m^3^) is derived from the values of *LAI*, tree and row spacings (*D_tree_* and *D_row_*, both in m) and a constant leaf area density (*LAD*, m^2^ m^-3^) given as input to the simulation:

$V={LAI D_{tree} D_{row}}/{LAD}$ (S118)

The value of *LAD* can either be set by the user or be given a default value of 1.5 m^2^ m^-3^. The latter was taken from experimental data by Villalobos et al. (1995) for olive cv. ‘Manzanilla’ olive trees. In the next step, the canopy is assumed to have a spheroidal shape, so *r_x_* can be deduced as:

$r_{x}=\sqrt[3]{{0.75 V}/\left( \pi R_{zx} \right)}$ (S119)

Where *R_zx_* is the ratio of the vertical to the horizontal canopy radius. Then, *GC* is calculated as:

$GC={\pi r_{x}^{2}}/\left( D_{tree} D_{row} \right)$ (S120)

With regard to fine roots, the value of *L_v_* for a given soil layer “*i*” in the soil zone “*j*” is deduced from the corresponding value of *B_froot_*:

$L_{v,i,j}={B_{froot,i,j} SRL}/\left( F_{j}{\Delta L}_{i,j} \right)$ (S121)

Where *SRL* is the specific root length (m g^-1^ DM) and *F_j_* is either *1-F_wet_* or *F_wet_* depending on whether the zone “*j*” is the dry or the wetted one, respectively. The former is treated as a parameter and its value was taken from measurements in a super-intensive olive cv. ‘Arbequina’ orchard performed by García-Tejera et al. (2017b).

## Soil carbon balance and heterotrophic respiration

The soil carbon balance and heterotrophic respiration are computed with an adaptation of the model proposed by Huang et al. (2009) and modified to take into account the effect of soil moisture on the rate of decomposition according to Verstraeten et al. (2006). The model considers three pools of carbon in the soil, represented by three state variables: the labile carbon pool (*CP_labile_*, g C m^-2^), which includes the fast decomposing carbon in plant residues; the resistant carbon pool (*CP_resistant_*, g C m^-2^), which includes slow decomposing carbon in plant residues; and the stable carbon pool (*CP_stable_*, g C m^-2^), which is the soil organic carbon resulting from the transformation of the latter due to the action of heterotrophic soil organisms. Inputs for *CP_labile_* and *CP_resistant_* include plant residues resulting from senescence (leaves and fine roots) and frost damage (leaves), although senescent leaves are only considered for the first layer of the soil. In addition, OliveCan presents a switch enabling the user to decide whether pruning residues should also be taken into account for the calculations for the first layer of soil or not (i.e. in case they are exported or burn).

For each tissue, the fraction of labile and resistant carbon is set as:

$F_{labile}={150+1.5N_{0}-0.57L_{0}}/{100}$ (S122)

Where *N_0_* and *L_0_* are the concentrations of nitrogen and lignin expressed in g kg^-1^ of dry matter that were taken from reports by Mariscal et al. (2000).

For each soil compartment, heterotrophic respiration (*R_H_*, g C m^-2^ d^-1^) is calculated as:

${RESP}_{H}={RESP}_{labile}+{RESP}_{stable}+\left( 1-0.375 \right){RESP}_{resistant}$ (S123)

Where the coefficient 0.375 represents the fraction of the resistant carbon consumed by soil organisms that is transformed into stable carbon (the value is taken directly from Huang et al. (2009)) the remaining variables (i.e. *RESP_labile_*, *RESP_stable_*, *RESP_resistant_*, g C m^-2^ d^-1^) are the respiration rates of the labile, stable and resistant carbon forms, respectively. Those are calculated assuming that they act as a first order chemical reaction modified by some environmental conditions:

${RESP}_{i}={kmax}_{i} F_{temp} F_{clay} F_{pH} F_{SW} {CP}_{i}$ (S124)

Where the sub-index ‘*i*’ refers to either labile, resistant or stable forms, *kmax_k_* is the kinetic constant of the reaction and *F_temp_*, *F_clay_*, *F_pH_* and *F_SW_* are dimensionless factors modulating the effect of soil temperature, clay content, pH and water content on the activity of heterotrophic organisms, respectively.

The factor *F_temp_* is calculated according to Huang et al. (2009):

$F_{temp}=Q_{10}^{\left( T-10 \right)/{10}}$ (S125)

Where *T* is daily soil temperature (ºC), assumed uniform within the soil profile and equal to the average air temperature and *Q_10_* is the relative increase of *kmax_i_* for a temperature increase of 10 ºC in relation to a reference temperature of 10 ºC.

The effect of soil pH on the activity of heterotrophic organisms is calculated as (Groenendijk and Kroes, 1997):

$F_{pH}=\frac{1}{1+exp\left[ -2.5\left( pH-5 \right) \right]}$ (S126)

The effect of soil structure on the activity of heterotrophic organisms is calculated as a function of the fraction of clay in the soil ([*clay*]) (Huang et al., 2009):

$F_{clay}=1-0.26\left[ clay \right]$ (S127)

The combined effect of water content and aeration is calculated according to Verstraeten et al. (2006) for every soil compartment as a function of factors related to soil aeration stress (*SAS*) and soil strength stress (*SSS*):

$F_{SW}=F_{SW,min}+\left( 1-SAS \right) SSS \left( F_{SW,max}-F_{SW,min} \right)$ (S128)

Where *F_SW,max_* and *F_SW,min_* are parameters defining the maximum and minimum values that *F_SW_* can reach, respectively. *SAS* increases with water content as the air content decreases and is calculated as:

$SAS=\left\{ \begin{aligned} 1-\left( \theta_{sat}-\theta\right)/{\left( \theta_{sat}-\theta_{crit} \right) if \theta>0.95 \theta_{sat}} \\ 0 if \theta\leq{0.95 \theta}_{sat} \end{aligned} \right.$ (S129)

SSS is calculated as a function of the relative water content in the soil:

$SSS=BDF \sin\frac{\theta-\theta_{LL}}{\theta_{UL}-\theta_{LL}}$ (S130)

Where *BDF* is a dimensionless factor depending on the apparent density of the soil (Jones et al., 1991). Values for *kmax_i_* where taken directly from Huang et al. (2009), *Q_10_* was taken from Raich and Schlesinger (1992) and *F_SW,max_* and *F_SW,min_* were taken from Verstraeten et al. (2006).

## Carbon exchange of the orchard

The model calculates both gross primary production (*GPP*, g CO_2_ m^-2^ d^-1^) and ecosystem respiration (*RESP_eco_*, g CO_2_ m^-2^ d^-1^) of the olive orchard as:

$GPP=A^{'}+{A'}_{fruit}$ (S131)

${RESP}_{eco}=\sum_{i} {RESP}_{M,i}+\sum_{i} {RESP}_{g,i}+{RESP}_{H}$ (S132)

Where the sub-index ‘*i*’ refers to the different tree organs. The net ecosystem exchange (*NEE*, g CO_2_ m^-2^ d^-1^) of the orchard is then deduced as:

$NEE=GPP-{RESP}_{eco}$ (S133)

# Management

In OliveCan, four management operations are considered: tillage, irrigation, harvest and pruning.

## Tillage

This management operation has an impact on the calculation of runoff and infiltration (Section 2.2). Whenever a user-defined tillage date (*DOY_tillage_*) is reached, the value of *SumE_rain_* is re-set to 0. In other words:

$\left\{ \begin{aligned} SumE_{rain}=SumE_{rain}+E_{rain} \mathrm{if} DOY \neq{DOY}_{tillage} \\ SumE_{rain}=0 \mathrm{if} DOY={DOY}_{tillage} \end{aligned} \right.$ (S134)

Note that, for non-tilled soils, the model skips Eq. S11.

## Irrigation

Any irrigation event provides a new source of water reaching the wetted soil zone and must be considered for the calculation of infiltration (Section 2.2). In terms of implementation, the user is required to define explicitly the irrigation amounts (*Irr*, mm) and dates (*DOY_irr_*). Alternatively, the model presents a routine which, at fixed periods between irrigations (*IrrInt*, d) calculates *Irr* as a fraction (*F_supply_*, dimensionless) of the cumulative *ET* lost since the last irrigation, provided a customizable irrigation season starting and ending at the dates *DOY_start,irr_* and *DOY_end,irr_*, respectively.

## Harvest

The harvest operation stores the value of *B_fruit_* into an output variable representing the dry yield of the orchard (*Y_dry_*, g DM m^-2^ ground) and then set both *B_fruit_* and *FrStage* to zero. For triggering the operation, the user is only required to define the day of harvest (*DOY_harvest_*). The model also calculates then the oil yield (*Y_oil_*, g oil m^-2^) as:

$Y_{oil}=F_{oil/DM}Y_{dry}$ (S135)

Where *F_oil/DM_* is the coefficient relating the oil content per gram of dry matter in the fruit. Its value is assumed constant and equal to 0.42 in our simulations, which was measured by López-Bernal et al. (2015) in an experiment conducted over three years in an olive cv. ‘Arbequina’ orchard.

## Pruning

This management operation leads to a reduction in the biomass of leaves, shoots and branches. The user is required to define the date of pruning (*DOY_prune_*) and the pruned fraction (*F_prune_*, dimensionless), which is defined as:

$F_{prune}={{LAI}_{cut}}/{LAI}$ (S136)

Where *LAI_cut_* (m^2^ leaf m^-2^ ground) is the leaf area index that is removed by pruning. Then, the model computes the removed biomass of shoots and branches by applying the same fraction and updates the corresponding states variables:

${LAI}_{{DOY}_{prune}+1}={LAI}_{{DOY}_{prune}}\left( 1-F_{prune} \right)$ (S137)

$B_{leaf,{DOY}_{prune}+1}={{LAI}_{{DOY}_{prune}+1}}/{SLA}$ (S138)

$B_{shoot,{DOY}_{prune}+1}=B_{shoot,{DOY}_{prune}}\left( 1-F_{prune} \right)$ (S139)

$B_{branch,{DOY}_{prune}+1}=B_{branch,{DOY}_{prune}}\left( 1-F_{prune} \right)$ (S140)

Finally, the model takes into account the biomass removed by pruning in the calculation of leaf senescence (Section 3.9) and conversion of shoots into branches (Section 3.10); and, at user’s choice, considers pruning residues to play a role for the computation of the soil carbon balance.

# Disaggregation of daily weather data

In the following, the algorithms used to disaggregate the daily weather data are described. The inputs to these algorithms were total daily solar radiation (MJ m^–2^ d^–1^), daily maximum temperature (°C), daily minimum temperature (°C) and daily average vapour pressure (kPa). The outputs of these algorithms, which can be calculated at any time resolution, are total solar radiation and PAR (W (m ground)^–2^) separated into direct and diffuse components as required by the SPAC model, air temperature (°C) and vapour pressure deficit (kPa). The reliability of the algorithms has been previously tested by comparing their outputs with a number of weather datasets recorded at a resolution of 10 min for several years (data not shown).

## Solar radiation

Daily values of solar radiation were decomposed into diurnal courses of direct and diffuse PAR using the algorithms described by Morales et al. (2016), just requiring the day of the year (*DOY*, 1 = 1^st^ January, 365 = 31^st^ December) and the latitude of the orchard (*LAT*, rad) as inputs. The equations calculate incoming solar radiation and incoming photosynthetically active radiation (PAR), as well as its diffuse and direct components. It is based on the algorithms proposed by Spitters et al. (1986) with some modifications in the notation introduced by Goudriaan and van Laar (1994). The modifications simplify the expressions and facilitate the comparison between the more comprehensive approach of Spitters et al. (1986) and the simpler approach of Goudriaan and van Laar (1994), which can be derived as particular case of the former method. Thus, the equations presented in this section should not be compared directly with the equations in Spitters et al. (1986) and, specifically, the parameters *a_1_* and *a_2_* in Equation S137 in this document are not related to the parameters *a* and *b* used in equation 5 in the original publication by Spitters et al. (1986).

In order to calculate the diurnal distribution of solar radiation, the angle between the sun and the horizon, known as the solar elevation angle (*β_s_*, rad), is needed. The calculation of this angle only requires knowing the values of *LAT*, *DOY* and the time of the day (*t*, 0 = midnight, 12 = solar noon). The solar elevation angle is calculated as:

$\sin\beta_{s}=a_{1}+a_{2}\cos\left( 2\pi\left( t-12 \right)/{24} \right)$ (S141)

Where *a_1_* = sin *LAT* sin *δ_s_*, *a_2_* = cos *Lat* cos *δ_s_* and *δ_s_* is the declination angle (rad). The declination angle changes during the year and it is caused by the orbiting movement of the Earth and the fact that the rotation axis is not perpendicular to the plane of the orbit. For each day of the year, the declination angle can be calculated as:

$\sin\delta_{s}=-\sin\left( {23.45\pi}/{180} \right)\cos\left( {2\pi\left( DOY+10 \right)}/{365} \right)$ (S142)

Note that *a_1_*, *a_2_*, and *δ_s_* are assumed constant within a given day. The time between sunrise and sunset (*DL*, h) is calculated as

$DL=t_{ss}-t_{sr}=12\left( 1+\frac{2}{\pi}\mathrm{asin}\left( {a_{1}}/{a_{2}} \right) \right)$ (S143)

Where *t_sr_* and *t_ss_* are the times of sunset and sunrise (h) and asin represents the arc-sine trigonometric function. The amount of solar radiation incident on a horizontal plane, before entering the atmosphere, is known as the extra-terrestrial solar radiation (*I_0_*, W m^–2^) and can be calculated as:

$I_{0}=I_{k}\sin\beta_{s}\left( 1+0.033 \cos\left( \frac{2\pi}{365} \right) \right)$ (S144)

Where *I_k_* is the solar constant (1367 W m^–2^) and the third factor in equation S139 describes the effect of the eccentricity of the Earth's orbit, which modifies the solar constant within the range 1320 – 1410 W m^–2^. The extra-terrestrial solar radiation received on a given day (*I_0,D_*, J m^–2^ ground day^–1^) is obtained from the integration of equation S139, whose analytical solution is known to be:

$I_{0,D}=\int_{0}^{24} I_{0} \sin\beta_{s}dt=I_{k}\left( 1+0.033 \cos\left[ \frac{2\pi\left( DOY-1 \right)}{365} \right] \right)3600\left( \sin LAT \sin\delta_{s} DL+\frac{24}{\pi}\cos LAT \cos\delta_{s}\sqrt{1-\frac{{\sin LAT \sin\delta_{s}}^{2}}{{\cos LAT \cos\delta_{s}}^{2}}} \right)$ (S145)

The ratio between the solar radiation on the surface of the Earth and the extra-terrestrial solar radiation is known as the atmospheric transmission coefficient. The daily atmospheric transmission (*τ_D_*, dimensionless) is defined as:

$\tau_{D}=\frac{I_{G,D}}{I_{O,D}}$ (S146)

Where *I_G,D_* is the daily solar radiation (J m^–2^ d^–1^). Assuming that the instantaneous transmission coefficient is constant during the day (*τ* = *τ_D_*), the instantaneous solar radiation of the surface of the Earth (*I_G_*, W m^–2^ ground) can be calculated as:

$I_{G}=I_{0}\frac{I_{G,D}}{I_{0,D}}=I_{G,D}\frac{\sin\beta_{s}}{\int_{0}^{24} \sin\beta_{s}dt}=I_{G,D}\frac{\sin\beta_{s}}{3600\left( a_{1} DL+\frac{24}{\pi} a_{2}\sqrt{1-{a_{1}^{2}}/{a_{2}^{2}}} \right)}$ (S147)

However, empirical observations indicate that the instantaneous transmission coefficient is higher around solar noon than after sunrise or before sunset. This decrease of transmission towards the extremes of the day is caused by the increase in the optical thickness of the atmosphere (as solar beams have to travel a longer path through the atmosphere) and the occurrence of meteorological phenomena such as fog and low clouds (Spitters et al., 1986). A simple way to describe this variation is by introducing an additional dependency on the solar angle (1 + *a_3_* sin*β*):

$I_{G}=I_{G,D}\frac{\sin\beta_{s}\left( 1+a_{3}\sin\beta_{s} \right)}{\int_{0}^{24} \sin\beta_{s}\left( 1+a_{3}\sin\beta_{s} \right)dt}=I_{G,D}\frac{\sin\beta_{s}\left( 1+a_{3}\sin\beta_{s} \right)}{3600\left( DL\left( a_{1}+a_{3}\left( a_{1}^{2}+0.5a_{2}^{2} \right) \right)+\frac{24}{\pi} a_{2}\left( 1+1.5a_{1} a_{3} \right)\sqrt{1-{a_{1}^{2}}/{a_{2}^{2}}} \right)}$ (S148)

Using Eqs. S136-S138 and S143, the daily total solar radiation can be disaggregated into the instantaneous solar radiation at any time of the day. The parameter *a_3_* was set to 0.4 in accordance to Morales et al. (2016). To calculate how much radiation is absorbed by the canopy, one needs to separate the solar radiation into its direct and diffuse components. The diffuse component is the fraction of solar radiation that is scattered by the particles in the atmosphere. The variations in the diffuse fraction of radiation are determined mainly by degree of cloudiness, which itself can be estimated by the atmospheric transmission coefficient. A first diffuse fraction can be calculated from the daily transmission coefficient. However, depending on the size of the scattering particles relative to the wavelength of the radiation being scattered, two types of scattering can be distinguished:

1. Mie scattering is produced by particles of a size in the order of magnitude of the wavelength being scattered. In the atmosphere, dust and aerosols are responsible for Mie scattering of solar radiation. This scattering is not perfectly isotropic as the intensity of the scattered radiation is higher in the direction in which the radiation propagates.

2. Rayleigh scattering is produced by particles of a size much smaller than the wavelength being scattered. In the atmosphere, gas molecules are responsible for Rayleigh scattering of solar radiation. This scattering is perfectly isotropic but the intensity of the scattered radiation decreases with the wavelength.

The importance of these modifications to the spectral and spatial component of diffuse radiation depends on the solar elevation angle and the degree of cloudiness. The scattering of light by clouds is the result of multiple refractions and reflections of light as it interacts with water droplets and ice crystals.

#### 5.1.1. Effect of cloudiness

The relationship between the daily diffuse fraction of radiation and atmospheric transmission coefficient is very similar for different locations of the Earth (Spitters et al., 1986) and can be described as:

$f_{D}=\frac{I_{df,D}}{I_{G,D}}=\left\{ \begin{matrix} 1 & \mathrm{if}\tau_{D}<0.07 \\ 1-2.3\left( \tau_{D}-0.07 \right)^{2} & if 0.07\leq\tau_{D}<0.35 \\ 1.33-1.46\tau_{D} & if 0.35\leq\tau_{D}<0.75 \\ 0.23 & if 0.75\leq\tau_{D} \end{matrix} \right.$ (S149)

where *I_df,D_* is the daily diffuse solar radiation (J m^–2^ day^–1^). Whereas the instantaneous transmission coefficient changes during the day as described (implicitly) by Eq. S145, the ratio *I_df_*/*I_0_*, where *I_df_* is the instantaneous diffuse solar radiation (W m^–2^), is assumed to be constant during the day, that is, *I_df,D_*/*I_0,D_* = *I_df_*/*I_0_*. This simplification is based on the fact that the reflection and absorption of solar radiation by the clouds and other scatters will tend to compensate the increase in the diffuse fraction. Thus, the diurnal variation of the diffuse fraction of solar radiation (*I_df_*/*I_G_*) is determined by *τ* i.e. *f_D_* = *I_df,D_*/(*τ I_0,D_*)). This means that the fraction of diffuse radiation is higher after sunrise and before sunset (low *τ*) and lower in the central hours of the day (high *τ*). If the day being simulated is characterized by intermittent cloudiness, the true diurnal evolution of *f_D_* may not follow this pattern.

#### 5.1.2. Effect of Mie scattering

In order to take into account the effect of Mie scattering (i.e. higher diffuse radiation in the direction of the sun), the additional radiation corresponding to this effect is calculated, subtracted from the diffuse solar radiation and added to the direct solar radiation. This way, the model can still assume an isotropic distribution of diffuse radiation, which simplifies the calculations, but the effect of Mie scattering is considered. The new corrected daily diffuse radiation (*I'_df,D_*, J m^–2^ day^–1^) is calculated as:

${I'}_{df,D}=\frac{I_{df,D}}{1+\left( 1-\left( \frac{I_{df,D}}{I_{G,D}} \right)^{2} \right)\cos^{2}\left( \frac{\pi}{2}-\bar{\beta}_{s} \right)\cos^{3}\left( \bar{\beta}_{s} \right)}$ (S150)

Where $\bar{\beta_{s}}$ is the average daily solar elevation angle. This correction is especially important in days with low cloudiness and intermediate solar elevation angles. The maximum correction is a reduction of 15%, obtained on a day without clouds and a daily average solar elevation of 45°. The mean daytime solar elevation angle may be calculated by integrating numerically Eq. S137 between sunrise and sunset. As a reference, given a latitude of 37.5° North, the daytime average solar elevation varies from 18.6° up to 40.9° throughout the year.

#### 5.1.3. Effect of Rayleigh scattering

The photosynthetically active radiation (PAR) is assumed to represent 50% of the solar radiation (*I_G_*), but the scattering due to the Rayleigh effect is higher for PAR than for solar radiation. Weighting by the effect of the different wavelengths on photosynthesis, Spitters et al. (1986) proposed to use the following expression:

$\frac{{PAR}_{df,D}}{{PAR}_{G,D}}=\left( 1+0.3\left( {1-\left( \frac{I_{df,D}}{I_{G,D}} \right)}^{2} \right) \right)\frac{{I'}_{df,D}}{I_{G,D}}$ (S151)

Where the subscripts for PAR have the same meaning as for solar radiation. The correction due to Mie scattering reduces the diffuse fraction but the correction due to Rayleigh scattering increases it and the result is that the diffuse fraction of PAR is always higher than the uncorrected fraction calculated with Eq. S143. This increase is higher for clear-sky days and for low and high solar angles.

## Air temperature

The variation of air temperature during the day is calculated as a function of the daily maximum (*T_max_*, ºC) and minimum (*T_min_*, ºC) temperatures recorded by a weather station. To do so, the following assumptions:

1. Minimum temperature occurs at sunrise
2. During the daytime, temperature follows a third order polynomial with a maximum occurring *τ_t_* hours after solar noon:

- $T=T_{min}+a_{t}t_{d}-b_{t}{t_{d}}^{2}-c_{t}{t_{d}}^{3}$ (S152)
- Where *t_d_* is time (h) after sunrise.

1. During the night, crop temperature decreases as a function of the square root of time after sunset. This is based on the simple model of nocturnal cooling by Brunt (1932):

- $T=T_{ss}-k_{n}\sqrt{t-DL}$ (S153)
- Where *T_ss_* is temperature at sunset (ºC) and *k_n_* is an empirical cooling coefficient.

The values of the coefficients in the two previous equations are obtained by solving the system of equations considering the following constraints:

- The derivative of *T* (Eq. S148) is zero at time *DL*/*2*+ *τ_t_*, where *τ_t_* represents the lag between solar noon and the time for which the maximum temperature is reached. In OliveCan a value of 2.5 h is adopted.
- Maximum temperature occurs at time *DL*/*2*+ *τ_t_*
- At sunset, the derivative of both Eqs. S148 and S149 are the same
- At sunrise, *T_min_* is equal to the result of Eq. S149.

The resolution of the system leads to:

$a_{t}=2\tau_{x}b_{t}+3\tau_{tx}^{2}c_{t}$ (S154)

$b_{t}=\left( \delta_{t}-2\tau_{tx}^{3}c_{t} \right)/{\tau_{tx}^{2}}$ (S155)

$c_{t}=\frac{\delta_{t}\left( \alpha_{t2}-2\alpha_{t1} \right)/\left( \tau_{tx}^{2} \right)-\delta_{t}^{'}}{3\alpha_{t1}\tau_{tx}^{2}+2\tau_{tx}\left( \alpha_{t2}-4\alpha_{t1} \right)-\alpha_{t3}}$ (S156)

$T_{ss}=T_{min}+a_{t}DL-b_{t}{DL}^{2}-c_{t}N^{3}$ (S157)

$k_{n}={{\delta^{'}}_{t}+a_{t}DL-b_{t}{DL}^{2}-c_{t}{DL}^{3}}/\sqrt{24-DL}$ (S158)

Where:

$\delta_{t}=T_{max}-T_{min}$ (S159)

${\delta^{'}}_{t}=T_{max}-{T_{min}}^{'}$ (S160)

$\alpha_{t1}=DL+2\sqrt{DL} \sqrt{24-DL}$ (S161)

$\alpha_{t2}={DL}^{2}+4 DL \sqrt{DL} \sqrt{24-DL}$ (S162)

$\alpha_{t3}={DL}^{3}+6 {DL}^{2} \sqrt{DL} \sqrt{24-DL}$ (S163)

$\tau_{tx}=\tau_{t}+{DL}/2$ (S164)

Where *T_min_’* is the minimum temperature (ºC) of the following day.

## Vapour pressure deficit

It is assumed that air vapour pressure (*e_a_*, kPa) is constant during the day, as diurnal fluctuations of this variable are generally small (Goudriaan and van Laar, 1994). The vapour pressure deficit (*VPD*, kPa) is calculated as:

$VPD=e_{s,a}-e_{a}$ (S165)

Where *e_s,a_* is the vapour pressure of saturated air (kPa). The vapour pressure deficit in air increases with air temperature, as hotter air is able to store more water vapour. In the range of 0-50 °C and when the main source of water vapour is liquid water, this increase can be computed as (Villalobos et al., 2016b):

$e_{s,a}=0.611exp\left( {17.3T}/\left( 237.3+T \right) \right)$ (S166)

Where *T* is the temperature of the air (°C), calculated as described in the previous sub-section.

# Requirements and initialization of simulations

In order to run simulations with OliveCan, the user requires to:

- Provide a weather dataset
- Set initial values for the state variables
- Set values for the soil, management and orchard parameters specific of the olive orchard to be simulated
- Provide a calendar of irrigation operations when required

## Weather dataset

OliveCan uses daily values of the following weather variables:

- Maximum air temperature (*T_max_*, ºC)
- Minimum air temperature (*T_min_*, ºC)
- Solar radiation (*I_G,D_*, MJ m^–2^ d^–1^)
- Precipitation (*P*, mm)
- Average wind speed (*U*, m s^-1^)
- Average vapour pressure (*e_a_*, kPa)

Apart from the whole seasons to be simulated, the weather dataset must include, at least, the temperature data since September 1^st^ (Northern hemisphere) or March 1^st^ (Southern hemisphere) in order to compute the initial phenological state (*FlStage*).

## Initialization of state variables

Some of the state variables in the model are difficult to measure or information about them might not be available. If such is the case, the model provides the initial value of *B_leaf_*, *B_shoot_*, *B_branch_*, *B_croot_* and *B_froot_* as a function of some more-easily estimable traits. To do so:

- The user must enter the (initial) values of *GC* and *H_tree_*. Then, the equations described in Section 3.13 are used to deduce *V*, *LAI* and *B_leaf_*.
- *B_shoot_* is assumed equal to *B_leaf_*
- Based on observations in one of the experiments of Pérez-Priego et al. (2014), *B_branch_* is calculated as a function of the age of the trees (*Age*, years) as:
- $B_{branch}=267 Age$ (S167)
- *B_croot_* is calculated as:
- $B_{croot}=0.2 B_{branch}$ (S168)
- *B_G,res_* is calculated as:
- $B_{G,res}=0.05 \left( B_{leaf}+B_{shoot}+B_{branch}+B_{croot} \right)$ (S169)
- The user must provide the distribution of *L_v_* values within the soil profile. Then Eq. S121 is used to deduce *B_froot_* for each soil compartment.
- Provided the dry yield of the year preceding the simulation, the fruit number for such year is calculated by dividing dry yield by the average single fruit dry mass (*SFM*), which is treated as a parameter. In the absence of yield data, *B_fruit_* is considered null at the start of the simulation. Moreover, for the computation of *FN* in the years to simulate, the model assumes that the number of fruits in the year preceding simulations (*FN_1_*) was null, whereas the variable *FN_max,1_* is calculated as:
- ${FN}_{max,1}={0.25 LAI}/{A_{leaf}}$ (S170)

With regard to phenology, the model assumes that *FrStage* and *VStage* are set to zero and *FlStage* is computed as a function of the temperature records in the autumn preceding the first year to simulate (as pointed above). Finally, the user must indicate the initial values of soil water content.

## Input parameters

The complete list of input parameters required for performing simulations with OliveCan is provided in Table S1. These parameters can be classified in different groups:

- Soil parameters: they depend on the type of soil. Some of them can be either measured or defined as a function of soil type using available literature. For instance, Campbell and Norman (1998) provide values for the parameters *Ψ_e_*, *b* and *k_sat_* for different soils.
- Orchard parameters: they depend on intrinsic characteristics of the olive orchard to be simulated such as location, canopy shape or planting density.
- Management parameters: they trigger management operations.
- Environment: this category includes the CO_2_ concentration in the air (*C_a_*). For present conditions, a value of 400 µmol CO_2_ mol^-1^ can be adopted.
- Simulation settings: this group only involves the parameter *N*, which is the number of periods in which the day is divided for the sub-day model computations regarding chilling accumulation, photosynthesis, root water uptake and maintenance respiration. The higher the value of *N*, the higher reliability of the model results concerning such processes and the higher computation time required. Allowing the user to set *N* is hence useful because compromise between reliability and computation time can be achieved. In any case, it is advisable to keep *N* above 24, in order to ensure numerical convergence.

## Irrigation data

When simulating irrigated olive orchards, the model requires either:

1. the dates in which irrigation is applied (*DOY_irr_*, day of year) and the amounts (*Irr*, mm) applied in each date if a particular irrigation calendar is to be simulated or
2. the dates delimiting the irrigation season (*DOY_start,irr_* and *DOY_end,irr_*, days of year), a fixed interval between irrigation events (*IrrInt*, d) and the fraction of the *ET* lost since the last irrigation to be applied (*F_supply_*, dimensionless)

# Parameter values

The complete list of parameters (excluding those that must be specifically entered by the user and presented in the previous section) including their dimensions, description and the values used in the simulations is provided in Table S2.

# References

Abdel-Razik, M. (1989). A model of the productivity of olive trees under optional water and nutrient supply in desert conditions. *Ecol. Modell.* 45, 179–204.

Allen, R.G., Pereira, J.S., Raes, D., Smith, M. (1998). *Crop evapotranspiration: guidelines for computing crop water requirements*. Rome: Vol. 56, Food and Agriculture Organization of the United Nations.

Barranco, D., Ruiz, N., Gómez-del Campo, M. (1995). Frost tolerance of eight olive cultivars. *HortScience* 40, 558-560.

Bernacchi, C.J., Singsaas, E.L., Pimentel, C., Portis, A.R., Long, S.P. (2001). Improved temperature response functions for models of Rubisco-limited photosynthesis. *Plant Cell Environ*. 24, 253-259.

Bonachela, S., Orgaz, F., Villalobos, F.J., Fereres, E. (2001). Soil evaporation from drip irrigated olive orchards. *Irrig. Sci*. 20, 65-71.

Bristow, K.L., Campbell, G.S., Calissendorff, C. (1984). The effects of texture on the resistance to water-movement within the rhizosphere. *Soil Sci. Soc. Am. J*. 48, 266-270.

Bustan, A., Avni, A., Lavee, S., Zipori, I., Yeselson, Y., Schaffer, A., Riov, J., Dag, A. (2011). Role of carbohydrate reserves in yield production of intensively cultivated oil olive (*Olea europaea* L.) trees. *Tree Physiol.* 31, 519–530.

Campbell, G.S. (1985). *Soil physics with BASIC: transport models for soil-plant systems*. Amsterdam: Elsevier.

Campbell, G.S., Norman, J.M. (1998). *Introduction to environmental biophysics*. New York: Springer.

Cuevas, J., Rallo, L., Rapoport, H.F. (1994). Initial fruit set at high temperature in olive, *Olea europaea* L. *J. Hortic. Sci*. 69, 665-672.

De Melo-Abreu, J.P., Barranco, D., Cordeiro, A.M., Tous, J., Rogado, B.M., Villalobos, F.J. (2004). Modelling olive flowering date using chilling for dormancy release and thermal time. *Agric. For. Meteorol.* 125, 117–127.

Díaz-Espejo, A., Walcroft, A.S., Fernández, J.E., Hafridi, B., Palomo, M.J., Girón, I.F. (2006). Modeling photosynthesis in olive leaves under drought conditions. *Tree Physiol*. 26, 1445–56.

Farquhar, G.D., von Caemmerer, S., Berry, J.A. (1980). A biochemical model of photosynthetic CO_2_ assimilation in leaves of C3 species. *Planta* 90,78–90.

Fernández, J.E., Giron, I.F., Moreno, F. (2001). Heat-pulse measurements of sap flow in olives for automatic irrigation: tests, root flow and diagnosis of water stress. *Agric. Water Manag*. 51, 99-123.

García-Tejera, O., López-Bernal, A., Villalobos, F.J., Orgaz, F., Testi, L. (2016). Effect of soil temperature on root resistance: implications for different trees under Mediterranean conditions. *Tree Physiol*. 36, 469-478.

García-Tejera, O., López-Bernal, A., Testi, L., Villalobos, F.J. (2017a). A soil-atmosphere continuum (SPAC) model for simulating tree transpiration with a soil multi-compartment solution. *Plant Soil* 412, 215-233.

García-Tejera, O., López-Bernal, A., Testi, L., Villalobos, F.J. (2017b). Analysing the interactions between wetted area and irrigation volume in an olive orchard using a SPAC model with a multi-compartment soil solution. *Irrig. Sci*. doi:10.1007/s00271-017-0549-5.

Gardner, W.R. (1960). Dynamic aspects of water availability to plants. *Soil Sci*. 89, 63-73.

Gómez, J.A., Giráldez, J.V., Fereres, E. (2001). Rainfall interception by olive trees in relation to leaf area. *Agric. Water Manag*. 49, 65-76.

Goudriaan, J., van Laar, H.H. (1994). *Modelling potential crop growth processes: textbook with exercises*. Kluwer Academic Publisher.

Groememdijk, P., Kroes, J.G. (1997). Modelling the nitrogen and phosphorus leaching to groundwater and surface water. DLO Winand Staring Centre, Wageningen.

Hacke, U.G., Sperry, J.S., Wheeler, J.K., Castro, L. (2006). Scaling of angiosperm xylem structure with safety and efficiency. *Tree Physiol*. 26, 689-701.

Hartmann, H.T. (1953). Effect of winter chilling on fruitfulness and vegetative growth in the olive. *Proc. Am. Soc. Hort. Sci*. 62, 184-190.

Huang, Y., Yu, Y., Zhang, W., Sun, W., Liu, S., Jiang, J., Wu, J., Yu, W, Wang, Y., Yang, Z. (2009). Agro-C: a biogeophysical model for simulating the carbon budget of agroecosystems. *Agric. For. Meteorol.* 95, 203-223.

Jarvis, P.G., McNaughton, K.G. (1986). Stomatal control of transpiration: scaling up from leaf to region. *Adv. Ecol. Res.* 15, 1–49.

Jones, A.C., Bland, W.L., Ritchie, J.T., Williams, J.R. (1991). “Simulation of root growth” in *Modeling plant and soil systems*, eds. J. Hanks, J.T. Ritchie (Agronomy 31. American Society of Agronomy), 91-123.

Jones, C.S., Kiniry, J.R. (1986). CERES-Maize: a simulation model of maize growth and development. Texas A & M University Press, College Station.

Koubouris, G.C., Metzidakis, I.T., Vasilakakis, M.D. (2009). Impact of temperature on olive (*Olea europea* L.) pollen performance in relation to relative humidity and genotype. *Environ. Exp. Bot*. 67, 209-214.

López-Bernal, A., Alcántara, E., Testi, L., Villalobos, F.J. (2010). Spatial sap flow and xylem anatomical characteristics in olive trees under different irrigation regimes. *Tree Physiol*. 30, 1536-1544.

López-Bernal, A., Garcia-Tejera, O., Orgaz, F., Testi, L., Villalobos, F.J. (2014). “Olive bud dormancy is induced by low temperatures” in *Proceedings of the XIII^th^ Congress of the European Society for Agronomy* (Debrecen, Hungary, 25-29 August), 253-254.

López-Bernal, A., García-Tejera, O., Vega, V.A., Hidalgo, J.C., Testi, L., Orgaz, F., Villalobos, F.J. (2015). Using sap flow measurements to estimate net assimilation in olive tres under different irrigation regimes. *Irrig. Sci*. 33, 357-366.

López-Bernal, A., Villalobos, F.J., García-Tejera, O., Testi, L., Orgaz, F. (2017) Do olive vegetative buds undergo a real dormant state in Winter? *Acta Hortic.* 1160, 227-230.

Mariscal, M.J., Orgaz, F., Villalobos, F.J. (2000). Radiation-use efficiency and dry matter partitioning of a young olive (*Olea europaea*) orchard. *Tree Physiol*. 20, 65–72.

Morales, A., Leffelaar, P.A., Testi, L., Orgaz, F., Villalobos F.J. (2016). A dynamic model of potential growth of olive (*Olea europaea* L.). *Eur. J. Agron.* 74, 93-102.

Moriana, A., Villalobos, F.J., Fereres, E. (2002). Stomatal and photosynthetic responses of olive (*Olea europaea* L.) leaves to water deficits. *Plant Cell Environ*. 25, 395–405.

Nadezhdina, N., Nadezhdin, V., Ferreira, M.I., Pitacco, A. (2007). Variability with xylem depth in sap flow in trunks and branches of mature olive trees. *Tree Physiol.* 27, 105-113.

Newman, E.I. (1969). Resistance to water flow in soil and plant. I. Soil resistance in relation to amounts of root: theoretical estimates. *J. Appl. Ecol*. 6, 1-12.

Orgaz, F., Villalobos, F.J., Testi, L., Fereres, E. (2007). A model of daily mean canopy conductance for calculating transpiration of olive canopies. *Funct. Plant Biol*. 34, 178.

Palese, A.M., Nuzzo, V., Dichio, B., Celano, G., Romano, M., Xiloyannis, C. (2000). “The influence of soil water content on root density in young olive trees” in *III International Symposium on Irrigation of Horticultural Crops* (International Society for Horticultural Sciences), 329–336.

Pérez-Priego, O., Testi, L., Kowalski, A.S., Villalobos, F.J., Orgaz, F. (2014). Aboveground respiratory CO_2_ effluxes from olive trees (*Olea europaea* L.). *Agrofor. Syst*. 88, 245-255.

Priestley, C.A. (1977). The annual turnover of resources in young olive trees. *J. Hortic. Sci*. 52, 105–112.

Proietti, P. (1998). Gas exchange in senescing leaves of *Olea europaea* L. *Photosynthetica* 35, 579–587.

Proietti, P., Famiani, F., Tombesi, A. (1999). Gas exchange in olive fruit. *Photosynthetica* 36, 423-432.

Raich, J., Schlesinger, W. (1992). The global carbon dioxide flux in soil respiration and its relationship to vegetation and climate. Tellus 44B, 81-99.

Raupach, M.R. (1994). Simplified expressions for vegetation roughness length and zero-plane displacement as functions of canopy height and area index. *Bound. Layer Meteorol.* 71, 211-216.

Ritchie, J.T. (1998). “Soil water balance and plant water stress” in *Understanding options for agricultural production*, eds. G.Y. Tsuji, G. Hoogenboom, P.K. Thornton (Springer Netherlands, Dordrecht), 41-54.

Romero, P., Castro, G., Gómez, J.A., Fereres, E. (2007). Curve number values for olive orchards under different soil management. *Soil Sci. Soc. Am. J*. 71, 1758-1769.

Ryan, M.G. (1990). Growth and maintenance respiration in stems of *Pinus contorta* and *Picea engelmannii*. *Can. J. For. Res.* 20, 48–57.

Scariano, L., Lo Bianco, R., Di Marco, L., Policarpo, M. (2008). “Dynamics of dry matter partitioning in young ‘Nocellara del Belice’ olive trees” in *Proceedings of the Fifth International Symposium on Olive Growing*, ed. M.T. Ozkaya, 397–401.

Soil Conservation Service (1985). “Estimation of direct runoff from storm rainfall” in *Hydrology. Natl. Eng. Handbk.*, Washington: USDA-SCS.

Sperry, J.S., Hacke, U.G., Pittermann, J. (2006). Size and function in conifer tracheids and angiosperm vessels. *Am. J. Bot*. 93, 1490-1500.

Spitters, C.J.T., Toussaint, H.A.J.M., Goudriaan, J. (1986). Separating the diffuse and direct component of global radiation and its implications for modeling canopy photosynthesis. Part I. Components of incoming radiation. *Agric. For. Meteorol*. 38, 217–229.

Steudle, E., Peterson, C.A. (1998). How does water get through roots? *J. Exp. Bot.* 49, 775-788,

Testi, L., Villalobos, F.J., Orgaz, F., Fereres, E. (2006). Water requirements of olive orchards: I simulation of daily evapotranspiration for scenario analysis. *Irrig. Sci*. 24, 69-76.

Trentacoste, E.R., Puertas, C.M., Sadras, V.O. (2012). Modelling the intraspecific variation in the dynamics of fruit growth, oil and water concentration in olive (*Olea europaea* L.). *Eur. J. Agron*. 38, 83–93.

Tuzet, A., Perrier, A., Leuning, R. (2003). A coupled model of stomatal conductance, photosynthesis and transpiration. *Plant Cell Environ.* 26, 1097-1116.

Tyree, M.T., Ewers, F.W. (1991). The hydraulic architecture of trees and other woody plants. *New Phytol*. 119, 345-360.

Tyree, M.T., Zimmermann, M.H. (2002). *Xylem structure and the ascent of sap*. Heidelberg: Springer-Verlag.

Verhoef, A., McNaughton, K.G., Jacobs, A.F.G. (1997). A parameterization of momentum roughness length and displacement height for a wide range of canopy densities. *Hydrol. Earth Syst. Sci*. 1, 81-91.

Verstraeten, W., Veroustraete, F., Feyen, J. (2006). On the temperature and water limitation of net ecosystem productivity: Implementation in the C-Fix model. *Ecol. Modell.* 199, 4-22.

Villalobos, F.J., Orgaz, F., Mateos, L. (1995). Non-destructive measurement of leaf area in olive (*Olea europaea* L.) trees using a gap inversion method. *Agric. For. Meteorol.* 73, 29–42.

Villalobos, F.J., Orgaz, F., Testi, L., Fereres, E. (2000). Measurement and modeling of evapotranspiration of olive (*Olea europaea* L.) orchards. *Eur. J. Agron*. 13, 155–163.

Villalobos, F.J., Testi, L., Fereres, E. (2016a). “The water budget” in *Principles of agronomy for sustainable agriculture*, eds. F.J. Villalobos, E. Fereres. (Cham: Springer), 91-106.

Villalobos, F.J., Mateos, L., Testi, L., Fereres, E. (2016b). “Air temperature and humidity” in *Principles of agronomy for sustainable agriculture*, eds. F.J. Villalobos, E. Fereres. (Cham: Springer), 55-67.

Villalobos, F.J., Testi, L., Hidalgo, J., Pastor, M., Orgaz, F. (2006). Modelling potential growth and yield of olive (*Olea europaea* L.) canopies. *Eur. J. Agron*. 24, 296–303.

Wheeler, J.K., Sperry, J.S., Hacke, U.G., Hoang, N. (2005). Inter-vessel pitting and cavitation in woody *Rosaceae* and other vesselled plants: a basis for a safety versus efficiency trade-off in xylem transport. *Plant Cell Environ*. 28, 800-812.

# Supplementary Figures and Tables

## Supplementary Figures

**Supplementary Figure 1.** Relational diagram showing a simple overview of the OliveCan components. Boxes represent the main modules of the model (water and carbon balances) and external data providers (weather and management). The main processes simulated in each module are listed inside the corresponding boxes. Some management operations have impacts on the water balance module (tillage and irrigation) whereas others affect the carbon balance module (harvest and pruning). Arrows between boxes represent communication between components. Daily weather data are used by the two main modules. Besides, some of the simulated processes (those including an * symbol) are simulated at customizable sub-day time steps, requiring disaggregation of the daily weather data.

**Supplementary Figure 2.** Shadow projected by an spheroid crown and overlapping by first-order neighbors within the row.

## Supplementary Tables

**Supplementary Table S1.** List of input parameters that must be defined according to soil type, orchard characteristics, management operations (excluding those related to irrigation) or user preferences prior to running simulations with OliveCan.

| **Parameter** | **Units** | **Description** |
| --- | --- | --- |
| SOIL | | |
| *b* | dimensionless | Shape factor for the calculation of soil water potential |
| *c_e_* | mm d^-0.5^ | Coefficient for calculating soil evaporation in stage II |
| [*clay*] | dimensionless | Clay content in the soil |
| *k_sat_* | kg s^-1^ m^-1^ kPa^-1^ | Soil saturated hydraulic conductivity |
| *n* | dimensionless | Number of soil layers |
| *pH* | dimensionless | Soil pH |
| *SHC* | dimensionless | Soil hydrological condition |
| *SWCON* | dimensionless | Fraction of soil water content above *θ_UL_* that drains on a period of one day |
| *U_e_* | mm | Amount of water to be evaporated from the soil to switch to stage II of soil evaporation |
| *ΔL* | m | Thickness of soil layer |
| *θ_LL_* | m^3^ m^-3^ | Water content at the permanent wilting point |
| *θ_sat_* | m^3^ m^-3^ | Water content at saturation |
| *θ_UL_* | m^3^ m^-3^ | Water content at field capacity |
| *Ψ_e_* | kPa | Air entry water potential |
| ORCHARD | | |
| *Age* | years | Age of the plantation at the start of simulations |
| *Alt* | m | Altitude of the orchard |
| *D_row_* | m | Row spacing |
| *D_tree_* | m | Tree spacing |
| *F_wet_* | dimensionless | Fraction of area wetted by localized irrigation |
| *Lat* | º | Latitude |
| *R_zx_* | dimensionless | Ratio of vertical to horizontal canopy radius |
| *LAD* | m^2^ m^-3^ | Leaf area density |
| MANAGEMENT | | |
| *DOY_harvest_* | dimensionless | Date (day of year) of harvesting |
| *DOY_prune_* | dimensionless | Pruning date (day of year) |
| *DOY_tillage_* | dimensionless | Tillage date (day of year) |
| *F_prune_* | dimensionless | Fraction of LAI to be removed by pruning |
| ENVIRONMENT | | |
| *C_a_* | µmol CO_2_ mol^-1^ | Atmospheric concentration of CO_2_ |
| SIMULATION SETTINGS | | |
| *N* | dimensionless | Number of periods in a day to be considered for the calculation of respiration, vernalization and SPAC model of root water uptake and assimilation |

**Supplementary Table S2.** List of parameters of OliveCan. Values shown correspond to those used in the simulations.

| **Parameter** | **Value** | **Units** | **Description** | **Source** |
| --- | --- | --- | --- | --- |
| *a* | -0.56 | h | Maximum loss of chilling hours caused by high temperatures during vernalization | De Melo-Abreu et al. (2004) |
| *a_3_* | 0.4 | dimensionless | Parameter that modifies the sinusoidal distribution of solar radiation during the day | Morales et al. (2016) |
| *Age_sapwood_* | 3 | year | Half of the age at which wood becomes inactive | Deduced from López-Bernal et al. (2010) |
| *A_leaf_* | 4.27 10^-4^ | m^2^ | Mean area of leaves | Villalobos et al. (2006) |
| *BDF* | 0.95 | dimensionless | Factor modulating the activity of heterotrophic organisms | Jones et al. (1991) |
| *c* | 1.25 | dimensionless | Ratio of vertical to horizontal fruit diameters | *Ad hoc* measurements |
| *C_alt_* | 0.7 | dimensionless | Fraction of fruit positions lost in a year preceded by other in which fruit load was maximum | Villalobos et al. (unpublished) |
| *cJ_cmax_* | 18.88 | dimensionless | Scaling constant for the Jcmax - temperature relationship | Díaz-Espejo et al. (2006) |
| *cKc* | *38.05* | dimensionless | Scaling constant for the Kc - temperature relationship | Díaz-Espejo et al. (2006) |
| *cK_o_* | 20.3 | dimensionless | Scaling constant for the Ko - temperature relationship | Díaz-Espejo et al. (2006) |
| *cR_d_* | *17.916* | dimensionless | Scaling constant for the Rd - temperature relationship | Díaz-Espejo et al. (2006) |
| *cV_cmax_* | *33.99* | dimensionless | Scaling constant for the Vcmax - temperature relationship | Díaz-Espejo et al. (2006) |
| *cΓ* | *19.02* | dimensionless | Scaling constant for the Γ - temperature relationship | Díaz-Espejo et al. (2006) |
| *F_FP_* | 2.4 | dimensionless | Ratio of fruits to leaf pairs | Villalobos et al. (unpublished) |
| *F_oil/DM_* | 0.42 | g oil g^-1^ DM | Ratio of oil to dry matter in the fruits | López-Bernal et al. (2015) |
| *F_SW,max_* | 1 | dimensionless | Maximum value for the factor of water and oxygen content in the soil | Verstraeten et al. (2006) |
| *F_SW,min_* | 0 | dimensionless | Minimum value for the factor of water and oxygen content in the soil | Verstraeten et al. (2006) |
| *FGR_max_* | 0.005 | g DM m^-2^ ground d^-1^ | Maximum daily growth rate of a fruit | Villalobos et al. (unpublished) |
| *g_0_* | 0.005 | µmol CO_2_ m^-2^ leaf s^-1^ | Night-time stomatal conductance (i.e. for zero gross assimilation) | García-Tejera et al. (2017a) |
| *HV* | 0.00028 | m^2^ sapwood m^-2^ leaf | Huber value | López-Bernal et al. (2010) |
| *kmax_labile_* | 2.6 10^-2^ | d^-1^ | Kinetic constant for the decomposition of labile carbon | Huang et al. (2009) |
| *kmax_resistant_* | 8.4 10^-4^ | d^-1^ | Kinetic constant for the decomposition of resistant carbon | Huang et al. (2009) |
| *kmax_stable_* | 6.8 10^-5^ | d^-1^ | Kinetic constant for the decomposition of stable carbon | Huang et al. (2009) |
| *K_res_* | 0.0167 | d^-1^ | Constant rate of reserve remobilisation | See Section 3.4 |
| *m* | 3.1 | dimensionless | Proportionality factor between photosynthesis and stomatal conductance | Moriana et al. (2002) |
| *PC_branch_* | 0.15 | dimensionless | Partitioning coefficient to branches | See Section 3.6 |
| *PC_croot_* | 0.2 | dimensionless | Partitioning coefficient to coarse roots | See Section 3.6 |
| *PC_froot_* | 0.3 | dimensionless | Partitioning coefficient to fine roots | See Section 3.6 |
| *PC_fruit,max_* | 1 | dimensionless | Maximum partitioning coefficient to fruits (source-limited) | Villalobos et al. (2006) and Morales et al. (2016) |
| *PC_leaf_* | 0.15 | dimensionless | Partitioning coefficient to leaves | See Section 3.6 |
| *PC_shoot_* | 0.2 | dimensionless | Partitioning coefficient to shoots | See Section 3.6 |
| *PV_branch_* | 0.65 | g DM g^-1^ G | Production value of branches | Mariscal et al. (2000) |
| *PV_croot_* | 0.65 | g DM g^-1^ G | Production value of coarse roots | Assumed same as branches |
| *PV_froot_* | 0.698 | g DM g^-1^ G | Production value of fine roots | Assumed same as shoots |
| *PV_fruit_* | 0.62 | g DM g^-1^ G | Production value of fruits | Mariscal et al. (2000) |
| *PV_leaf_* | 0.672 | g DM g^-1^ G | Production value of leaves | Mariscal et al. (2000) |
| *PV_shoot_* | 0.698 | g DM g^-1^ G | Production value of shoots | Mariscal et al. (2000) |
| *Q10* | 2.4 | dimensionless | Relative increase of the kinetic constant for a temperature increase of 10 ºC in relation to the reference temperature | Raich and Schlesinger (1992) |
| *r_M,branch_* | 1.13 10^-4^ | g C g^-1^ DM d^-1^ | Specific maintenance respiration coefficient for branches at 0 ºC | Pérez-Priego et al. (2014) |
| *r_M,croot_* | 1.13 10^-4^ | g C g^-1^ DM d^-1^ | Specific maintenance respiration coefficient for coarse roots at 0 ºC | Same as branches |
| *r_M,froot_* | 1.81 10^-3^ | g C g^-1^ DM d^-1^ | Specific maintenance respiration coefficient for fine roots at 0 ºC | Same as leaves |
| *r_M,fruit_* | 1.56 10^-3^ | g C g^-1^ DM d^-1^ | Specific maintenance respiration coefficient for fruits at 0 ºC | Pérez-Priego et al. (2014) |
| *r_M,leaf_* | 1.81 10^-3^ | g C g^-1^ DM d^-1^ | Specific maintenance respiration coefficient for leaves at 0 ºC | Pérez-Priego et al. (2014) |
| *r_M,shoot_* | 7.78 10^-5^ | g C g^-1^ DM d^-1^ | Specific maintenance respiration coefficient for shoots at 0 ºC | Same as branches |
| *r_root_* | 0.00016 | M | Mean diameter of fine roots | García-Tejera et al. (2017b) |
| *s_f_* | 0.0023 | kPa^-1^ | Coefficient in the model of Tuzet et al. (2003) modulating the sensitivity of stomatal conductance to leaf water potential | García-Tejera et al. (2017b) |
| *SFM* | 0.5 | g DM | Average dry mass for a single fruit | Villalobos et al. (unpublished) |
| *SLA* | 4.2 10^-3^ | m^2^ leaf g^-1^ DM | Specific leaf area | Villalobos et al. (2006) |
| *SRL* | 9 | m g^-1^ DM | Specific root length | García-Tejera et al. (2017b) |
| *SumDTBB* | 7 | d | Number of days above *TBB* required for bud break | Based on experiments by López-Bernal et al. (2014, 2017) |
| *SumTUV* | 156 | h | Chilling hours required to enter the dormant phase for vegetative growth | Based on experiments by López-Bernal et al. (2014, 2017) |
| *SumU* | 469 | h | Chilling hours required to enter the forcing phase in the reproductive buds | De Melo-Abreu et al. (2004) |
| *T_0_* | 7.3 | ºC | Optimal temperature for chilling accumulation | De Melo-Abreu et al. (2004) |
| *T_BB_* | 13 | ºC | Temperature threshold for bud break | Based on experiments by López-Bernal et al. (2014, 2017) |
| *T_b_* | 9.1 | ºC | Base temperature for calculation of thermal time | De Melo-Abreu et al. (2004) |
| *T_CD_* | -11.8 | ºC | Critical temperature below which frost damage results in the complete defoliation of the tree | Barranco et al. (2005) |
| *T_CH_* | 45 | ºC | Maximum daily temperature above which all fruits are removed as a consequence of extreme heat stress | Koubouris et al. (2009) |
| *T_ND_* | -9.7 | ºC | Temperature threshold below which frost damage occurs | Barranco et al. (2005) |
| *T_NH_* | 35 | ºC | Maximum daily temperature below which fruit production is not affected by heat stress | Koubouris et al. (2009) |
| *TT0* | 490 – 495 | ºC d | Thermal time requirement for flowering following the vernalization phase | De Melo-Abreu et al. (2004) |
| *TT1* | 300 | ºC d | Thermal time that must be accumulated since flowering for considering fruits as a sink of assimilates | See Section 3.2.3 |
| *TT2* | 2800 | ºC d | Thermal time requirement for reaching maturity | See Section 3.2.3 |
| *TT_HS1_* | 344 | ºC d | Thermal time to be accumulated for the beginning of the heat-sensitive time window for fruit production | See Section 3.12 |
| *TT_HS2_* | 638 | ºC d | Thermal time to be accumulated for the end of the heat-sensitive time window for fruit production | See Section 3.12 |
| *TT_res_* | 100 | ºC d | Thermal time since bud break for which reserve remobilization is considered | See Section 3.4 |
| *T_UV_* | 13.6 | ºC | Threshold temperature for chilling accumulation of vegetative organs | Based on experiments by López-Bernal et al. (2014, 2017) |
| *T_x_* | 20.7 | ºC | Chilling temperature at which chilling hours loss is maximum | De Melo-Abreu et al. (2004) |
| *VD* | 5 × 10^7^ | m^-2^ | Vessel density in the sapwood | López-Bernal et al. (2010) |
| *α_B_* | 2 | dimensionless | Value at which *θ/θ_sat_* equals *δ'* | Bristow et al. (1984) |
| *αF* | 0.2 | mol e^-^ mol^-1^ quanta | Low-light quatum yield of CO_2_ assimilation | Díaz-Espejo et al. (2006) |
| *β_B_* | 30 | dimensionless | Velocity at which *r_r_(T,θ)* approaches infinity | Bristow et al. (1984) |
| *β_R,branch_* | 0.0899 | ºC^−1^ | Coefficient for calculating maintenance respiration of branches as a function of temperature | Pérez-Priego et al. (2014) |
| *β_R,croot_* | 0.0899 | ºC^−1^ | Coefficient for calculating maintenance respiration of coarse roots branches as a function of temperature | Assumed same as branches |
| *β_R,froot_* | 0.0374 | ºC^−1^ | Coefficient for calculating maintenance respiration of fine roots as a function of temperature | Assumed same as leaves |
| *β_R,fruit_* | 0.0577 | ºC^−1^ | Coefficient for calculating maintenance respiration of fruits as a function of temperature | Pérez-Priego et al. (2014) |
| *β_R,leaf_* | 0.0374 | ºC^−1^ | Coefficient for calculating maintenance respiration of leaves as a function of temperature | Pérez-Priego et al. (2014) |
| *β_R,shoot_* | 0.1134 | ºC^−1^ | Coefficient for calculating maintenance respiration of shoots as a function of temperature | Pérez-Priego et al. (2014) |
| *δ_B_* | 0.25 | dimensionless | Critical value of *θ/θ_sat_* at which *r_r_(T,θ)* becomes limiting | Bristow et al. (1984) |
| *ΔH_a_J_cmax_* | 35350 | J mol^-1^ | Activation energy for *J_cmax_* | Díaz-Espejo et al. (2006) |
| *ΔH_a_K_c_* | *79430* | J mol^-1^ | Activation energy for *K_c_* | Díaz-Espejo et al. (2006) |
| *ΔH_a_K_o_* | *36380* | J mol^-1^ | Activation energy for *K_o_* | Díaz-Espejo et al. (2006) |
| *ΔH_a_R_d_* | *44790* | J mol^-1^ | Activation energy for *R_d_* | Díaz-Espejo et al. (2006) |
| *ΔH_a_V_cmax_* | *73680* | J mol^-1^ | Activation energy for *V_cmax_* | Díaz-Espejo et al. (2006) |
| *ΔH_a_Γ* | *37830* | J mol^-1^ | Activation energy for *Γ* | Díaz-Espejo et al. (2006) |
| *θ_crit_* | 0.25 | dimensionless | Threshold of relative water content below which root growth is affected | Jones and Kiniry (1986) |
| *θ_F_* | 0.9 | dimensionless | Degree of curvature of the parabola | Díaz-Espejo et al. (2006) |
| *ϕ* | 0.002 | m ºC^-1^ d^-1^ | Daily increase in root depth per accumulated degree day | Jones and Kiniry (1986) |
| *ϕ_v_* | 3.6 × 10^-5^ | m | Mean diameter of xylem vessels | López-Bernal et al. (2010) |
| *Ψ_f_* | -1000 | kPa | Coefficient in the model of Tuzet et al. (2003) representing the leaf water potential threshold below which stomatal closure is induced | García-Tejera et al. (2017b) |
